# Novel mutations of TCTN3/LTBP2 with cellular function changes in congenital heart disease associated with polydactyly

**DOI:** 10.1111/jcmm.15950

**Published:** 2020-10-24

**Authors:** Huan‐Xin Chen, Zi‐Yue Yang, Hai‐Tao Hou, Jun Wang, Xiu‐Li Wang, Qin Yang, Lin Liu, Guo‐Wei He

**Affiliations:** ^1^ Center for Basic Medical Research & Department of Cardiovascular Surgery TEDA International Cardiovascular Hospital, Chinese Academy of Medical Sciences & Peking Union Medical College Tianjin China; ^2^ College of Life Sciences Nankai University Tianjin China; ^3^ Zhejiang University Hangzhou Zhejiang China; ^4^ Drug Research and Development Center Wannan Medical College Wuhu Anhui China; ^5^ Department of Surgery Oregon Health and Science University Portland OR USA

**Keywords:** cellular function, congenital heart disease, gene mutation, LTBP2, polydactyl, TCTN3, transcriptomics

## Abstract

Congenital heart disease (CHD) associated with polydactyly involves various genes. We aimed to identify variations from genes related to complex CHD with polydactyly and to investigate the cellular functions related to the mutations. Blood was collected from a complex CHD case with polydactyly, and whole exome sequencing (WES) was performed. The CRISPR/Cas9 system was used to generate human pluripotent stem cell with mutations (hPSCs‐Mut) that were differentiated into cardiomyocytes (hPSC‐CMs‐Mut) and analysed by transcriptomics on day 0, 9 and 13. Two heterozygous mutations, LTBP2 (c.2206G>A, p.Asp736Asn, RefSeq NM_000428.2) and TCTN3 (c.1268G>A, p.Gly423Glu, RefSeq NM_015631.5), were identified via WES but no TBX5 mutations were found. The stable cell lines of hPSCs‐LTBP2^mu^/TCTN3^mu^ were constructed and differentiated into hPSC‐CMs‐LTBP2^mu^/TCTN3^mu^. Compared to the wild type, LTBP2 mutation delayed the development of CMs. The TCTN3 mutation consistently presented lower rate and weaker force of the contraction of CMs. For gene expression pattern of persistent up‐regulation, pathways in cardiac development and congenital heart disease were enriched in hPSCs‐CM‐LTBP2^mu^, compared with hPSCs‐CM‐WT. Thus, the heterozygous mutations in TCTN3 and LTBP2 affect contractility (rate and force) of cardiac myocytes and may affect the development of the heart. These findings provide new insights into the pathogenesis of complex CHD with polydactyly.

## 1 INTRODUCTION

Congenital heart disease (CHD) is the most common congenital anomaly with a worldwide occurring in about 9 per 1000 live births and accounts for nearly one‐third of all major congenital anomalies,^1^ and the prevalence may be up to 2~3%, if minor cardiac abnormalities such as bicuspid aortic valve are included.^2^ The advances in surgical management and longevity of patients have been improved. However, there is approximately 20% early mortality for the most complex cardiac defects and late mortality is a relatively common occurrence.^3^

The genetic factors associated with CHD remain challenging. We recently identified that mutations of TBX5,^4^ PLGL1^5^ and HOMEZ^6^ are associated with ventricular septal defect. Further, we also identified the prevalence of 22q11.2 variations in CHD.^7^

CHD associated with malformation of dactyl has a complex genetic basis. Among various syndromes and complexes, Holt‐Oram syndrome (HOS, OMIM 142900) is a recognized rare condition (1/100 000 live births) characterized by anterior pre‐axial limb and cardiac malformations including atrial septal defects (ASD), ventricular septal defect (VSD), or multiple and complex malformations.^8^ Mutations in TBX5 have been reported to cause autosomal dominant HOS.^9^ Studies have shown that the genetic heterogeneity in HOS may be caused by the mutations of different genes. Several genes, such as homeobox genes, peptide growth factors and retinoic acid receptors, have been proposed to contribute to cardiac and limb development and regarded as important candidate genes for HOS.^10^

To identify the genetic characteristics of complex CHD and polydactyly with emphasis on whether TBX5 was involved, we performed whole exome sequencing (WES) on blood sample of a patient with complex CHD and polydactyly who had successful surgical repair. On the basis of the findings of the mutations in a number of genes, we subsequently performed related cellular function studies.

## 2 MATERIALS AND METHODS

### 2.1 Whole Exome Sequencing (WES) study

**FIGURE 1:**
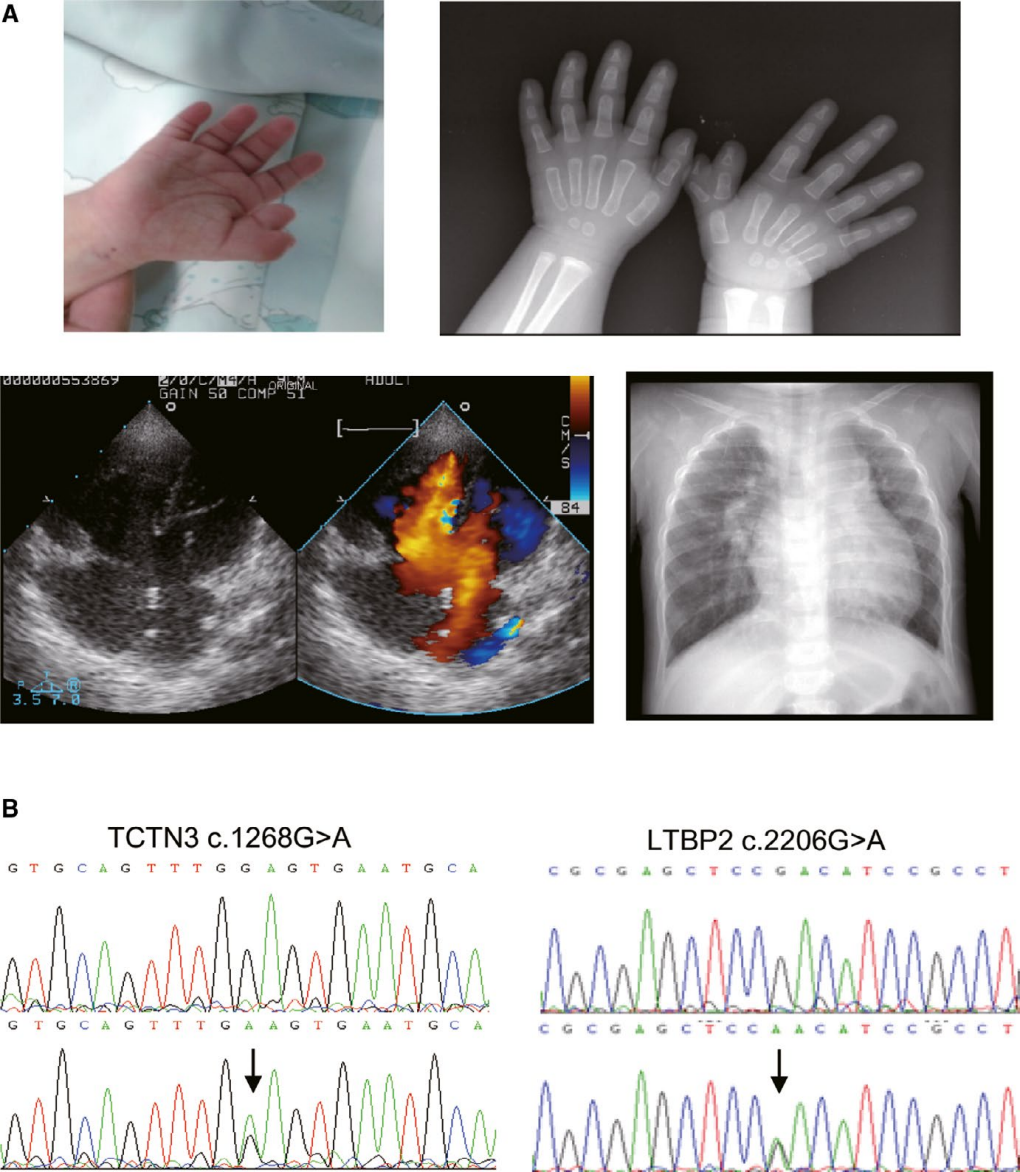
Typical phenotypes of the patient and potential pathogenic mutations. (A) Clinical features of the patient with both congenital heart defects and polydactyly. Atrioventricular septal defect (AVSD) was diagnosed by echocardiography; The X‐ray shows the enlarged heart shadow and the polydactyly. (B) Chromatograms of the two heterozygous mutations in the study. Top panels show wild type, and bottom panels show heterozygous mutations. Mutations are marked with arrows

Blood sample was obtained from the patient who was one‐and‐a‐half‐year‐old girl with complex CHD including complete atrioventricular septal defect (AVSD), patent ductus arteriosus (PDA), secondary atrial septal defect, pulmonary hypertension, and polydactyly (Figure 1A). Genomic DNA was extracted. WES was performed by BGI (Beijing Genomic Institute, Shenzhen, china), and the SNV/Indel was detected and analysed, using the UCSC hg19 human genome. The frequency of the variants in our study was filtered by population databases of dbSNP, 1000Genomes, ESP6500,gnomAD, and HGVD (BGI owned).

The study protocol was approved by the Institutional Review Board and Ethics Committee of TEDA international cardiovascular hospital.

Primers (Table S1) were designed to amplify, and the PCR products were sequenced by 3500 Genetic Analyzer (Applied Biosystems) to verify the results of WES.

### 2.2 Establishment of human pluripotent stem cells (hPSCs) with mutations

To verify the correlation between TCTN3 (RefSeq NM_015631.5)/LTBP2 (RefSeq NM_000428.2) mutation and clinical phenotype of the patient, the hPSCs with point mutation were established as model by CRISPR/Cas9‐ mediated genome engineering.

The plasmid pSpCas9 (BB)‐2A‐Puro (PX459) was a gift from Dr Feng Zhang's laboratory in MIT. Single‐guide RNAs (sgRNAs) were designed using online design tool (http://crispr.mit.edu). Pairs of oligos for target sequences were annealed and cloned into BbsI site of the Cas9 expression plasmid px459. Templates were generated by pEASY‐Blunt Simple Cloning Kit (CB111‐01, TransGen Biotech) according to the manufacturer's protocol. Null sense mutations on templates were generated to avoid double strands break by Cas9.

The hPSCs were cultured in Essential 8 medium (E8, A1517001, Thermo Fisher Scientific) without feeder layer cells. Culture dishes and plates were coated with Matrigel (356230, Corning) for one hour at 37°C incubator before seeding cells. The medium was changed daily, and cells were routinely passaged every 4 days. After 80% confluency was reached, the transfection was performed using the Human Stem Cell Nucleofector Kit 1 (VPH‐5012 Lonza) and the Amaxa Nucleofector Ⅱ according to the manufacturer's protocol.

About 24 hours after transfection, cells were selected by puromycin (2 μg/ml) for 6 hours. Then medium was changed to E8 medium daily for 5 days. PCR amplified was carried out using PrimeSTAR Max Premix (R045, TaKaRa), and the program consisted of an initial denaturation step 95°C for 5 minutes, followed by 35 cycles at 95°C for 30s, 58°C (LTBP2) or 62°C (TCTN3) for 30s and 72°C for 50s; the final extension at 72°C for 5 minutes. Positive clones were validated by Sanger sequencing and further cultured for following experiments. Primers used can be found in Table S1.

### 2.3 Detection of pluripotency markers in hPSCs

The hPSCs in experimental and control groups were collected and resuspended in cell lysis buffer. Total protein was extracted and measured by BCA Protein Assay Kit (23227, Thermo Fisher Scientific). Protein samples were electrophoresed on SDS‐PAGE gel, blotted onto PVDF membrane and probed with primary antibodies, including anti‐OCT4 (sc5279, Santa Cruz), anti‐Nanog (sc293121, Santa Cruz) and anti‐β‐actin (P30002, Abmart). Secondary antibodies conjugated with horseradish peroxidase (HRP) were used to detect the expression of the target protein.

Total RNA was extracted using TRIzol (15596018, Thermo Fisher Scientific) according to the manufacturer's protocol. The first‐strand cDNA was synthesized using random primers and M‐MLV Reverse Transcriptase (Invitrogen). Real‐time PCR was set up in duplicate with the Faststart Universal SYBR Green Master Mix (Roche) and carried out on an iCycler MyiQ2 Detection System (BIO‐RAD). Each sample was repeated 3 times and normalized using GAPDH as internal control. Relative quantitative evaluation of target gene was determined by comparing the threshold cycles. Primers (listed in Table S1) were confirmed for the specificity with dissociation curves.

The hPSCs were collected, preconditioned, and then blocked in 3% goad serum and 0.1% BSA in PBS. Samples were incubated with the primary antibodies overnight at 4°C, including anti‐OCT4, anti‐Nanog, anti‐SSEA4 (MAB4303, Millipore), and anti‐TNNT2. Goat Anti‐Mouse IgG (H+L) FITC (115‐095‐003; Jackson), Goat Anti‐Rabbit IgG (H+L) Alexa Fluor® 594 (111‐585‐003; Jackson) and Goat Anti‐Mouse IgM Alexa Fluor® 488(A‐21042; Invitrogen) were used as secondary antibodies. The cell nucleus was stained with DAPI. Fluorescence was digitally imaged on Carl Zeiss Axio‐Imager Z2 fluorescence microscope.

### 2.4 Differentiated hPSCs into cardiomyocytes (CMs)

The PSC Cardiomyocyte Differentiation Kit (A2921201, Thermo Fisher Scientific) was used for differentiation of hPSCs into CMs according to the manufacturer's protocol. Cell samples were collected on day 0, 9 and 13 during differentiation.

### 2.5 RNA‐sequencing and bioinformatics analysis

The CMs differentiated from hPSCs (hPSCs‐CMs) were cultured and harvested. A total amount of 3 mg of RNA per sample was extracted. The mRNA was enriched and fragmented into short fragments and was reverse transcripted into cDNA with random primers. Sequencing libraries were generated using NEBNext Ultra RNA Library Prep Kit for Illumina (NEB, United States) following manufacturer's recommendations and index codes were added to attribute sequences to each sample. The sequencing was performed by Gene Denovo Biotechnology Company (Guangzhou, China).

After filtering, clean reads were mapped to the human reference genome (GRCh38.p10). Gene abundances were quantified by RSEM. R software was used to perform principal component analysis (PCA) and identify differentially expressed genes (DEGs) between samples and control (http://www.r‐project.org/).

The analysis of DEGs expression pattern was performed to assess the gene expression trend during the development of myocardial cell, using Short Timeseries Expression Miner (STEM) software. Gene Ontology (GO) functions and KEGG (Kyoto Encyclopedia of Genes and Genomes) pathways enrichment analysis of DEGs were performed.

### 2.6 Statistical analysis

Data were analysed by ANOVA or *t* tests using StatView software from SAS Institute Inc (Cary, NC). The *P* value was calculated, and statistical significance was defined as *P* < 0.05(*), *P* < 0.01(**) or *P* < 0.001(***).

The more detailed methods are described in the Supplementary Method.

## 3 RESULTS

### 3.1 TCTN3 and LTBP2 mutations identified by WES

WES was performed on the patient with complex CHD, and the mean read depth of the target regions of each sample ranged from 80×. From whole exon regions of the 20,000 genes in human genome, 21 490 variants in 9005 genes were detected. By filtering based on allele frequency and function, 137 variants in 123 genes were finally identified (Table S2). In the 137 variants, four genes were found to be involved in both heart and dactyly. Those were LTBP2, TCTN3, CHD7 and TWIST1 (Table S2).

We did cellular function experiments for all these 4 genes. The two heterozygous mutations in exon 11 of TCTN3 (c.1268G>A, p.Gly423Glu, RefSeq NM_015631.5) and exon 12 of LTBP2 (c.2206G>A, p.Asp736Asn, RefSeq NM_000428.2) that were involved in both cardiac defects and polydactyly were successfully introduced into hPSCs by CRISPR/Cas9. The attempts to introduce mutations of CHD7(c.6571G>A, p.Glu2191Lys, RefSeq NM_017780.3) and TWIST1 (c.259_276delGCGGGCGGCGGCGGCGGC, p.Ala87_Gly92del, RefSeq NM_000474.3) into hPSCs by CRISPR/Cas9 were unsuccessful despite of multiple experiments.

TCTN3 c.1268G>A was not reported in population databases of dbSNP, 1000Genomes, and ESP6500. The allele frequency in HGVD (BGI) was 0.0015. The charge was changed by the mutation; glycine with uncharged polarity was substituted by glutamic and negative‐charged. Online software was used to explore the conservation and damaging. This mutation site is highly conserved in various vertebrates (PhyloP score: 3.121); SIFT (score: 0.01) and PolyPhen2 (score: 1) predicted TCTN3 c.1268G>A to be deleterious; MutationTaster predicted that the mutant was damaging; Human Splicing Finder showed that TCTN3 c.1268G>A broke an exon splicing enhancer and created an exon splicing silencer, thus potentially alter the splicing.

The allele frequency of LTBP2 c.2206G>A was 0.0009, 0.0035 and 0.0008 in population databases of dbSNP, 1000Genomes, and HGVD (BGI). The charge was changed by the mutation from negative‐charged to uncharged. PhyloP (score: 3.278) predicted that the site was highly conserved in vertebrates; SIFT (score: 0.02) and PolyPhen2 (score: 0.966) predicted the mutation to be deleterious; MutationTaster predicted that the mutant was damaging; Human Splicing Finder showed that the variant broke an exon splicing enhancer thus potentially alter the splicing. Sanger sequencing was used to validate these two mutations. Figure 1B shows the chromatograms of the mutations. Interestingly, there were no mutations found in the TBX5 gene in this patient.

### 3.2 The LTBP2/TCTN3 mutation was introduced separately into hPSCs by CRISPR/Cas9 system

To study the relationship between gene mutations and clinical phenotype of the patient, LTBP2/TCTN3 with point mutation (LTBP2 ^mu^/TCTN3^mu^) was introduced separately into hPSCs by CRISPR/Cas9 technology to observe the changes of cells.

**FIGURE 2:**
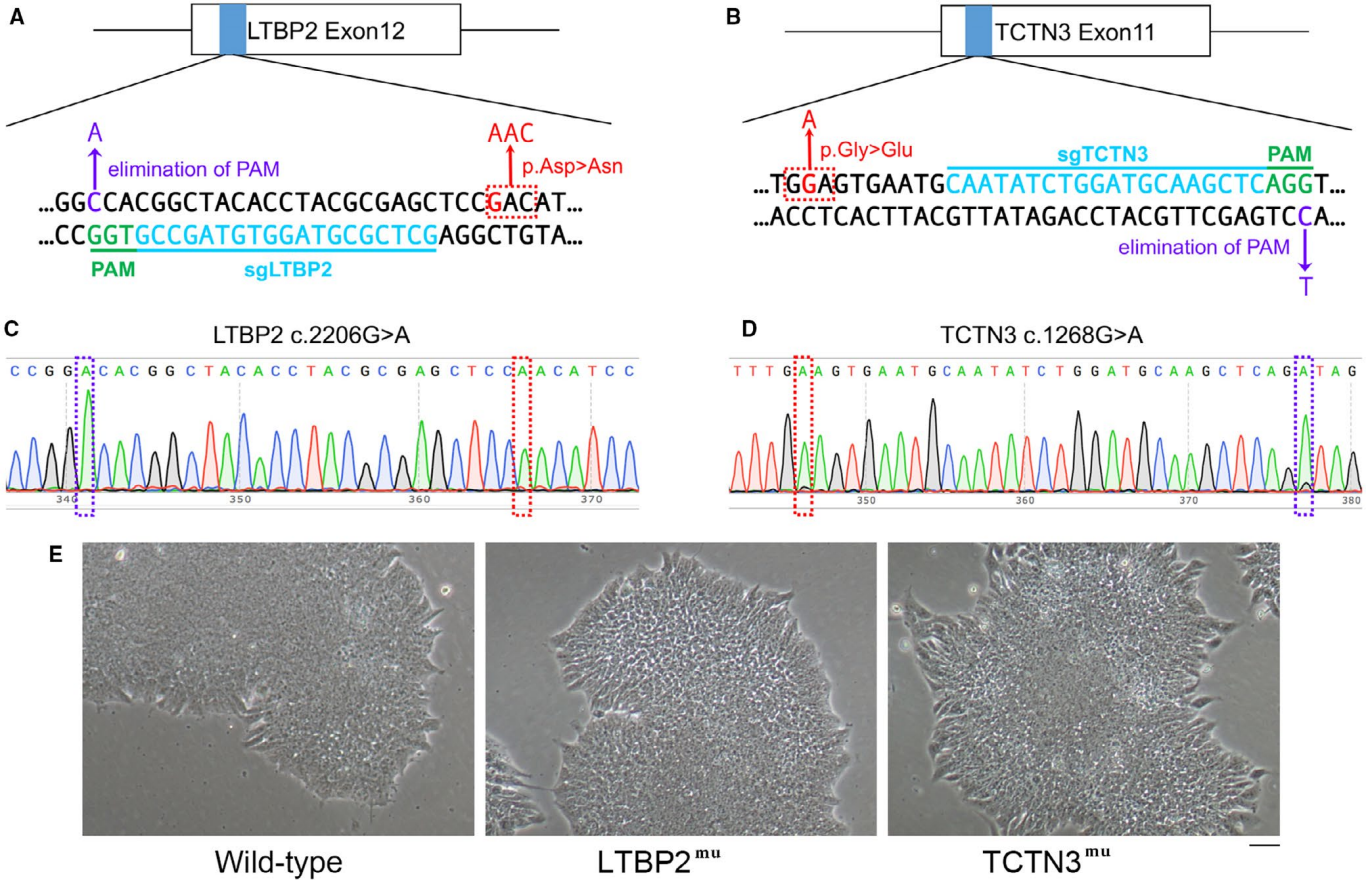
Generation of mutant cell lines. (A and B) Schematic of Cas9/sgRNA‐targeting sites in LTBP2 and TCTN3. The designed sgRNAs (sgLTBP2 and sgTCTN3) target sequences are underlined in blue colour; the protospacer‐adjacent motif sequences are highlighted in green colour; mutations are labelled in red colour; and synonymous mutations are marked in purple colour. (C and D) Mutations in positive cell lines were validated by Sanger sequencing. (E) Cell morphology of wild‐type and mutant hPSCs. Scale bar, 50 μm

Double‐strand DNA with LTBP2 ^mu^ or TCTN3^mu^ was generated as template to repair DNA double‐strand break. Synonymous mutations within protospacer‐adjacent motif (PAM) were introduced to avoid breaking the templates again (Figure 2A and B). Templates together with sgRNAs were co‐transfected into hPSCs WA26 (wild‐type). Sanger sequencing was used to validate homozygous mutations LTBP2 (c.2206G>A) and TCTN3 (c.1268G>A) in clones (Figure 2C and D). We successfully established two cell lines, hPSC‐LTBP2^mu^ and hPSC‐TCTN3^mu^. The cell morphologic changes were observed using inverted microscope and cells grew well in three groups (Figure 2E).

### 3.3 Detection of the developmental pluripotency of hPSCs

**FIGURE 3:**
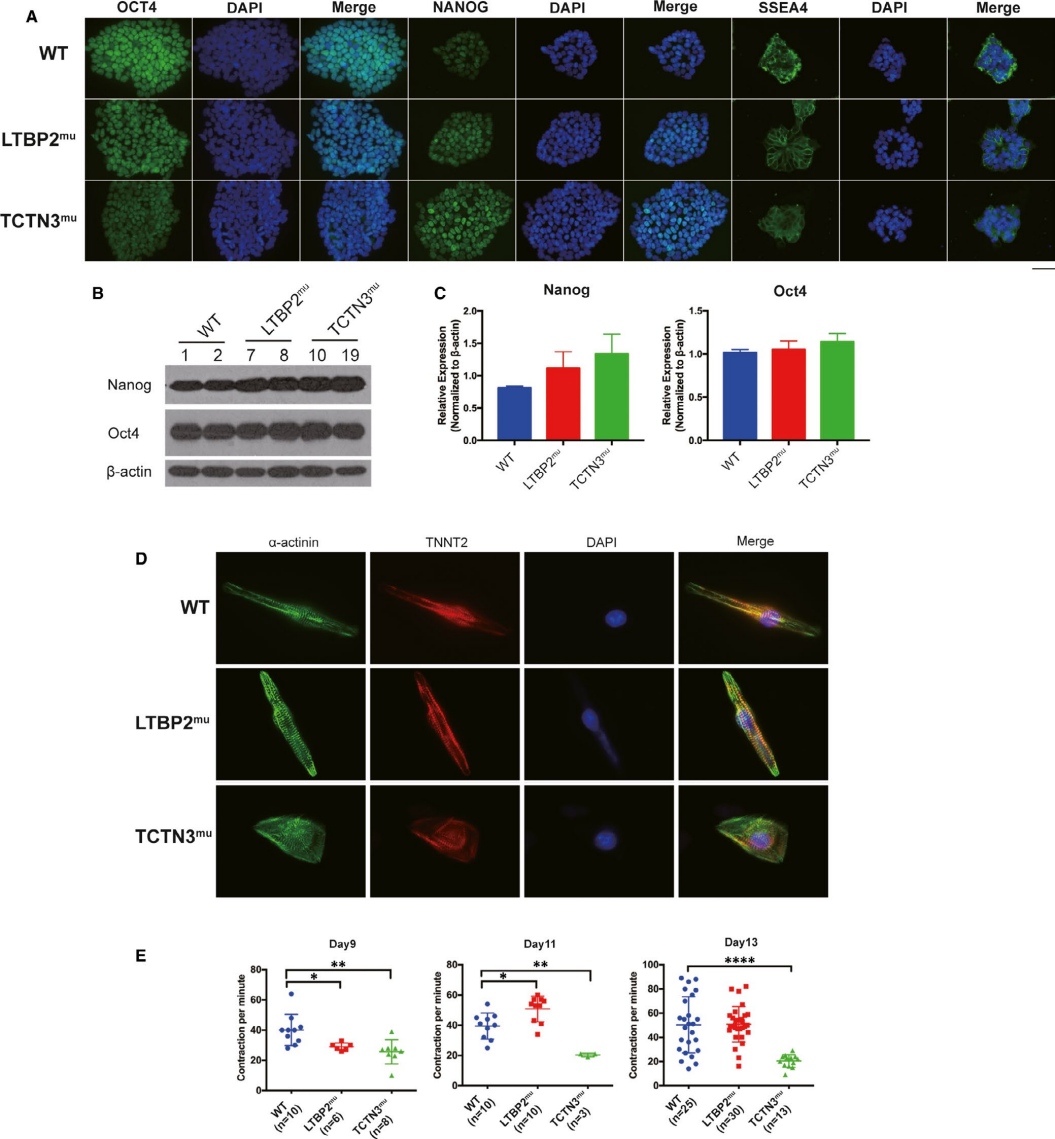
Pluripotency of hPSCs and contraction frequency of hPSC‐CMs were tested. (A) Expression of pluripotency markers (Oct4, Nanog and SSEA4) in hPSCs were detected by immunofluorescence. Scale bar, 50 μm. (B and C) Expression of Oct4 and Nanog was analysed by Western blot and Q‐PCR. (D) Immunofluorescence test for cardiomyocytes marker TNNT2. (E) Contraction rate of hPSC‐CMs on day 9, day 11 and day 13

The expression levels of important pluripotent markers Oct4, Nanog and SSEA4 were detected by immunofluorescence (IF) microscopy, Western bolt and Q‐PCR (Figure 3). The expression level of Nanog was WT<LTBP2^mu^ < TCTN3^mu^ with *P* value > 0.05 (Figure 3B and C); the other markers had no differences between the study groups (Figure 3A‐C). These results indicated that there were no significant differences between hPSCs‐LTBP2^mu^/TCTN3^mu^ and the wild type; these two mutations may not affect the developmental pluripotency of hPSCs.

### 3.4 Gene mutations changed the contraction frequency and cell differentiation of cardiomyocytes

To simulate the development of CMs, hPSCs were directly differentiated into CMs (hPSC‐CMs) in vitro. As CMs marker, TNNT2 was detected by immunofluorescence, indicating that cell differentiation was successful (Figure 3D).

During the process of differentiation, the contraction rate of cells was calculated (Figure 3E). The rate of group hPSC‐CMs‐LTBP2^mu^ was significantly slower than hPSC‐CMs‐WT on day 9 (Video S1, S2) and the rate increased on days 11‐13 (Video S3), suggesting that LTBP2^mu^ resulted in delayed development of hPSC‐CMs. Interestingly, the contraction of hPSC‐CMs‐TCTN3^mu^ was affected with significantly lower beating rate and weaker force (Video S4). These observations suggest that LTBP2^mu^ and TCTN3^mu^ affect cardiac rhythm and contraction of the heart.

### 3.5 Transcriptomics analysis of hPSC‐CMs

Transcriptomics analysis of group hPSC‐CMs‐LTBP2^mu^, hPSC‐CMs‐TCTN3^mu^, and hPSC‐CMs‐WT were performed using RNA‐sequencing (RNA‐Seq) on day 0, day 9 and day 13. The gene transcripts with the absolute value of log2FC>1 (minimal 2‐fold difference in expression) and FDR<0.05 (false discovery rate, adjusted *P*‐value threshold) were considered as significant DEGs.

**FIGURE 4:**
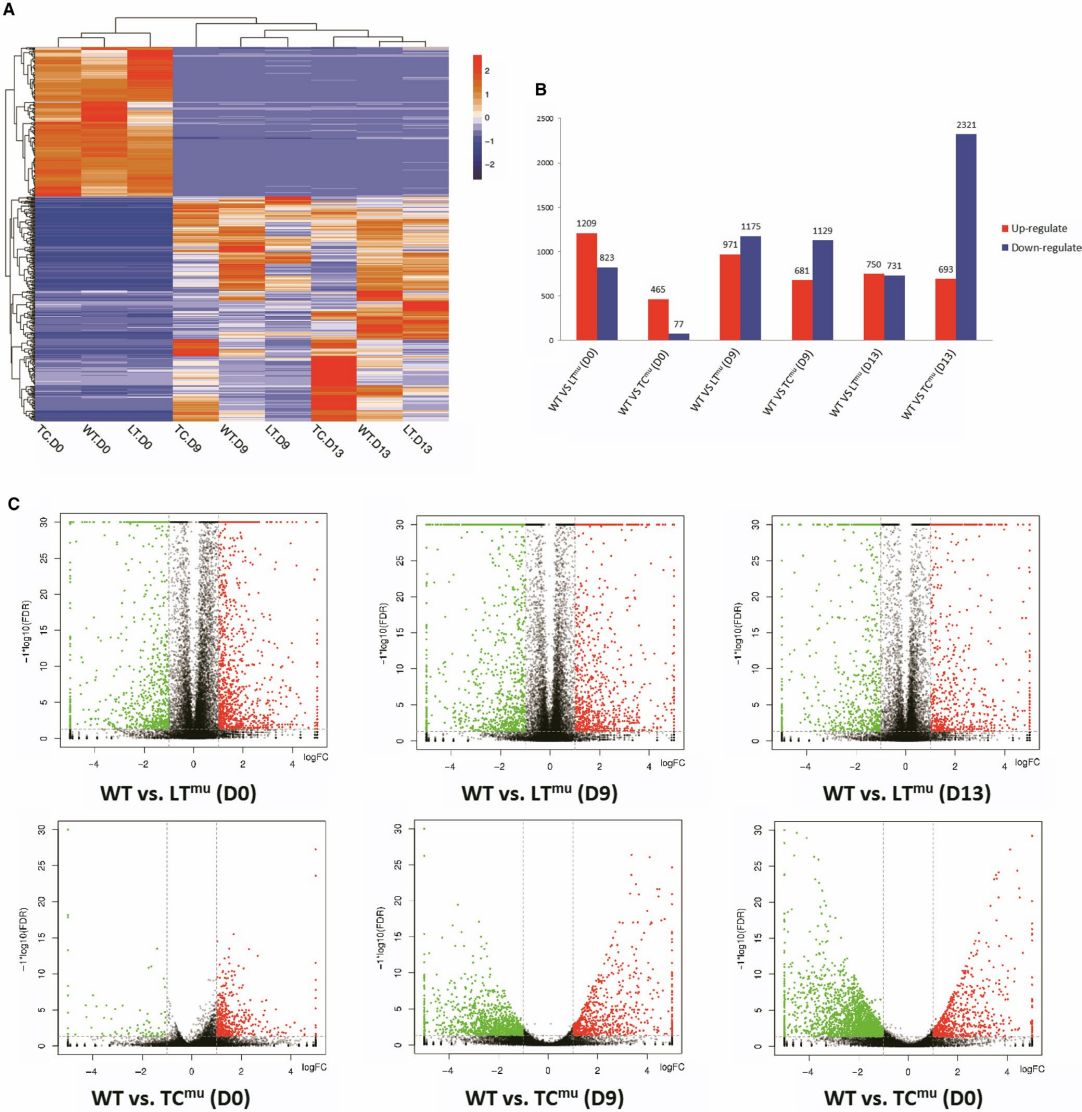
Overview of transcriptomics analysis. (A) Heatmap of top 500 DEGs in hPSC‐CMs‐WT/LTBP2^mu^/TCTN3^mu^ on days 0, 9 and 13. (B) Up‐regulated (red) and down‐regulated (blue) DEGs in hPSC‐CMs‐WT vs. hPSC‐CMs‐LTBP2^mu^, hPSC‐CMs‐WT vs. hPSC‐CMs‐ TCTN3^mu^ on days 0, 9 and 13. (C) Volcano plot shows gene expression in hPSC‐CMs‐LTBP2^mu^ and hPSC‐CMs‐TCTN3^mu^, compared with hPSC‐CMs‐WT on days 0, 9 and 13, based on a cut‐off value of 2‐fold differential expression and FDR < 0.05. The red (up‐regulated) and green (down‐regulated) dots represent DEGs; the black dots show non‐DEGs

Figure 4A shows the heatmap of top 500 DEGs in group hPSC‐CMs‐LTBP2^mu^, hPSC‐CMs‐TCTN3^mu^, and hPSC‐CMs‐WT on day 0, 9, 13. Genes with similar expression pattern in different samples were clustered together. Compared to hPSC‐CMs‐WT group, expression of various degrees of genes was up‐ or down‐regulated in hPSC‐CMs‐LTBP2^mu^ and hPSC‐CMs‐TCTN3^mu^. The most significant changes occurred in hPSC‐CMs‐TCTN3^mu^ group, 77 genes were down‐regulated in day 0 and 2321 genes were down‐regulated in day 13, compared with hPSC‐CMs‐WT (Figure 4B). Figure 4C represents the volcano plots for DEGs in hPSC‐CMs‐WT vs. hPSC‐CMs‐LTBP2^mu^ and hPSC‐CMs‐WT vs. hPSC‐CMs‐TCTN3^mu^ on different days respectively (|log2FC|> 1 and FDR<0.05).

### 3.6 Bioinformatics analysis of DEGs: expression pattern, function, and pathway

**FIGURE 5:**
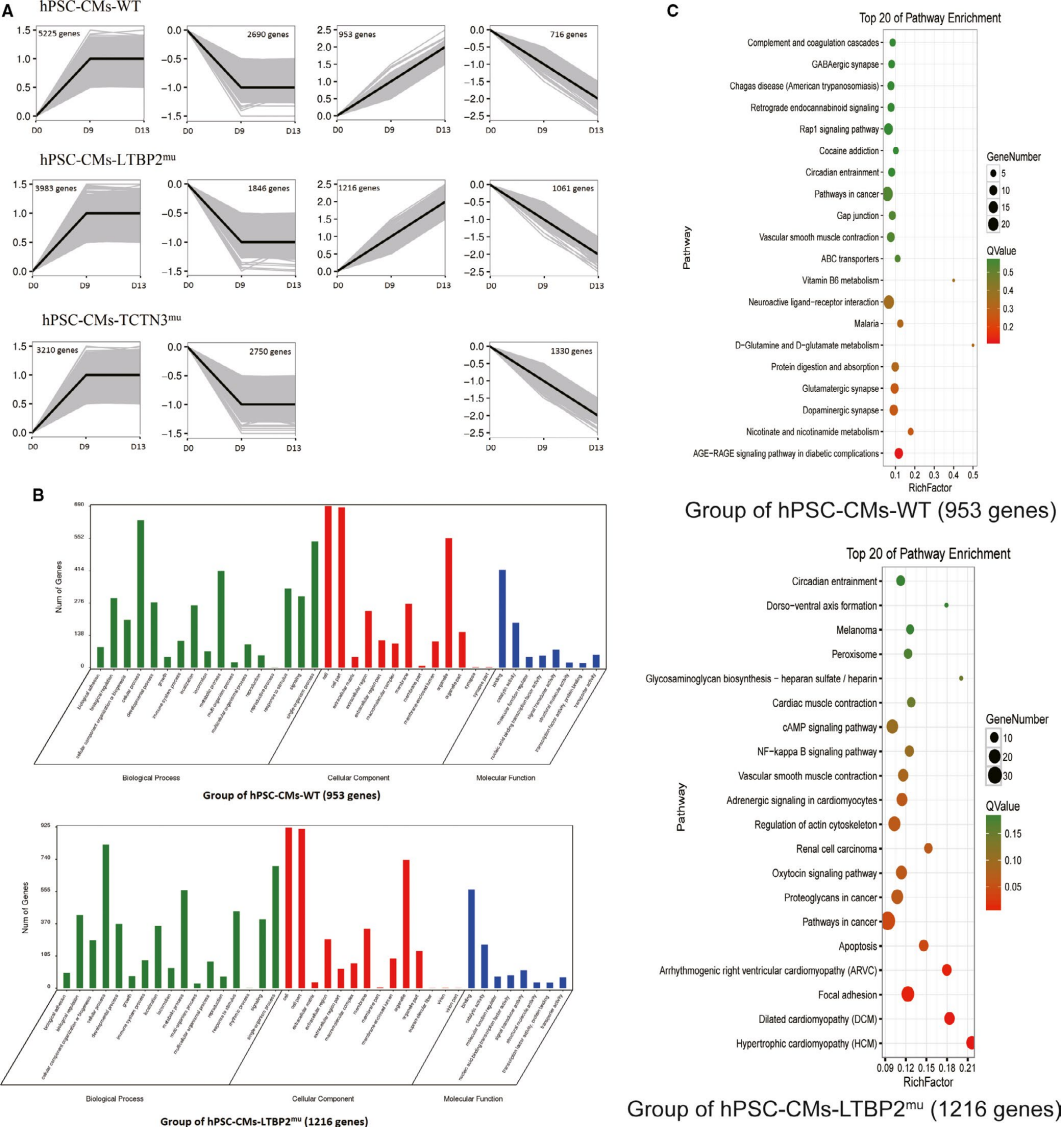
Bioinformatics analysis of DEGs: expression pattern, function and pathway. (a) Gene expression pattern analysis of DEGs. In each panel, the grey lines represent the expression pattern of each gene, and the black line represent the expression tendency of all the genes. The number of genes belonging to each pattern was labelled above the panel. The vertical axes represent relative expression levels (log2FC) across the developmental stages of cell differentiation. The horizontal axes indicate different times of differentiation at day 0, 9, and 13. (b) GO functional enrichment analysis of DEGs in hPSC‐CMs‐WT and hPSC‐CMs‐LTBP2^mu^. (c) Bubble chart for KEGG enrichment of DEGs between hPSC‐CMs‐WT and hPSC‐CMs‐LTBP2^mu^. Rich factor is a ratio of DEGs in pathway to all genes in the same pathway, greater the Rich factor, higher the degree of pathway enrichment. Q value range from 0 to 1, which is P value corrected for multiple‐hypothesis testing. Lower value indicates greater pathway enrichment

The DEGs of each group on different days were clustered based on gene expression pattern using STEM software. Figure 5A shows significant difference expression patterns (*P* < 0.05) in gene expression in groups of hPSC‐CMs‐WT, hPSC‐CMs‐LTBP2^mu^, and hPSC‐CMs‐TCTN3^mu^.

The genes of persistent up‐regulated expression pattern in hPSC‐CMs‐WT and hPSC‐CMs‐LTBP2^mu^ were analysed by GO and KEGG pathway enrichment. The DEGs were enriched and classified into 3 functional categories: biological process, cellular component, and molecular function (Figure 5B). Analysis of KEGG pathway enrichment showed that 238 pathways were enriched including 267 DEGs in hPSC‐CMs‐WT and 261 pathways were enriched including 370 DEGs in hPSC‐CMs‐LTBP2^mu^ (Table S3). Compared to hPSC‐CMs‐WT, some pathways in cardiac development and congenital heart disease were enriched in the top 20 pathways with the most significant P values, such as hypertrophic cardiomyopathy (HCM), dilated cardiomyopathy (DCM), arrhythmogenic right ventricular cardiomyopathy (ARVC), vascular smooth muscle contraction, and cardiac muscle contraction.

## 4 DISCUSSION

The present study demonstrates that in complex CHD associated with polydactyly the mutations of LTBP2 and TCTN3 may be potential pathological cause since these mutations are associated with changes of cellular functions that may affect the development of the heart. Further, this study again demonstrates that TBX5 mutations may not present in complex CHD associated with polydactyly.

As an important transcription factor, TBX5 regulates a wide variety of developmental processes. Particularly, TBX5 interacts with NKX2‐5 and GATA4 and co‐regulates cardiac gene expressions in heart development.^4^ In human, more than one hundred of TBX5 mutations identified are associated with HOS.^11^ Although it is well known that causal genes in HOS involve mutations in regulatory parts of TBX5, this syndrome may also involve other genes.^12^ Darwich and associate reported that KLF13 interacts physically and functionally with TBX5 to synergistically activate transcription of cardiac genes and therefore KLF13 may be a genetic modifier of the HOS gene TBX5.^13^ Another study identified a 14(q23.3∼24.2q31.1) deletion in a patient with HOS and suggested that chromosome 14 interstitial deletion is associated with clinical features of HOS.^14^ One of the aims of the present study was to explore whether there were mutations in TBX5 in this complex CHD with Polydactyly. Interestingly, there were no mutations found in TBX5. In view of the complexity of malformation of heart associated with dactyl, we searched Online Mendelian Inheritance in Man (OMIM) and Genetic Testing Registry (GTR) database of NCBI. Table 1 shows that there are various phenotypes that include both heart and dactyl malformation. Among those, only in HOS TBX5 mutations are present.

**TABLE 1:** The diseases associated with congenital malformation of heart and dactyl

Location	Phenotype	Phenotype MIM number	Inheritance	Gene	Gene MIM number	Clinical Features	
						Heart	Dactyl
1q22	Grange syndrome	602 531	AR	YY1AP1	607 860	congenital heart defects	brachysyndactyly
1q32.1	Orofaciodigital syndrome V	174 300	AR	DDX59	615 464	tetralogy of Fallot (TOF), ventricular septal defect	polydactyly
3pter‐p25	3p‐ syndrome	613 792	AD	‐	‐	congenital heart defects	polydactyly
4p16.2	Weyers acrofacial dysostosis	193 530	AD	EVC2	607 261	congenital heart disease	polydactyly
6p12.1‐ p11.2	Carpenter syndrome	201 000	AR	RAB23	606 144	congenital heart defects	brachydactyly, syndactyly, polydactyly
6q15	Cardiospondylocarpofacial syndrome	157 800	AD	MAP3K7	602 614	congenital mitral regurgitation	brachydactyly
6q25.3	Coffin‐Siris syndrome 1	135 900	AD	ARID1B	614 556	congenital heart defects	brachydactyly
7p21.1	Saethre‐Chotzen syndrome with or without eyelid anomalies	101 400	AD	TWIST1	601 622	cardiac malformation	brachydactyly, syndactyly
10q26.13	Saethre‐Chotzen syndrome	101 400	AD	FGFR2	176 943	cardiac malformation	brachydactyly, syndactyly
12p13.33	Timothy syndrome	601 005	AD	CACNA1C	114 205	congenital heart disease	syndactyly
12q24.21	Holt‐Oram syndrome	142 900	AD	TBX5	601 620	congenital heart lesion	syndactyly, polydactyly
15q15.1	Adams‐Oliver syndrome 6	616 589	AD	DLL4	605 185	congenital heart defects	syndactyly, brachydactyly, or oligodactyly
15q26.3	Weill‐Marchesani 4 syndrome, recessive	613 195	AR	ADAMTS17	607 511	cardiac defects or abnormal heart rhythm	brachydactyly
16q12.1	Townes‐Brocks syndrome 1	107 480	AD	SALL1	602 218	congenital heart disease	polydactyly, syndactyly,
17q24.3	Long QT syndrome 7	170 390	AD	KCNJ2	600 681	valvular heart disease	brachydactyly, syndactyly, clinodactyly.
19q13.2	Carpenter syndrome 2	614 976	AR	MEGF8	604 267	congenital heart disease	brachydactyly, syndactyly, polydactyly
20p12.2	McKusick‐Kaufman syndrome	236 700	AR	MKKS	604 896	congenital heart disease	polydactyly
20p12.3	Short stature, facial dysmorphism, and skeletal anomalies with or without cardiac anomalies	617 877	AD	BMP2	112 261	congenital heart defects	brachydactyly, clinodactyly
22q11.21	Velocardiofacial syndrome	192 430	AD	TBX1	602 054	heart malformation	polydactyly
22q11.21	DiGeorge syndrome	188 400	AD	TBX1	602 054	outflow tract defects of the heart	flexion contracture of all fingers
Chr.X	Orofaciodigital syndrome VIII	300 484	XLR	‐	‐	atrioventricular septal defect	postaxial polydactyly
Xp11.3	TARP syndrome	311 900	XLR	RBM10	300 080	congenital heart malformation	polydactyly, syndactyly
Xq26.2	Simpson‐Golabi‐Behmel syndrome, type 1	312 870	XLR	GPC3	300 037	congenital heart defects	polydactyly, syndactyly
Xq28	Otopalatodigital syndrome, type II	304 120	XLD	FLNA	300 017	heart malformation	syndactyly, clinodactyly

Abbreviations: AR, autosomal recessive; AD, autosomal dominant; DR, digenic recessive; XLR, X‐linked recessive; XLD, X‐linked dominant.

With respect to the major heart defect in this case, AVSD is a group of phenotypically and genetically heterogeneous heart malformations. Previous studies determined that there are two main causal genes CRELD1^15^ and N2RF2,^16^ which have been identified in numerous unrelated affected individuals with confirmed pathogenicity in animal models. Additional studies demonstrated that missense mutations in other genes are also associated with AVSD, including PTPN11, GATA4‐6, LEFTY2, FOXP1, ACVR2B, NODAL, ZIC3.^17^ However, there were no mutations found in these genes in the present case.

Polydactyly is one of the most common hereditary limb malformations and highly heterogeneous condition that depicts broad inter‐ and intra‐familial clinical variability. Previous studies have shown that mutations in GLI3^18^ and SHH^19^ are main genetic factors to cause polydactyly. These two genes play the key role in the control of digit number during limb development.^20^ Interestingly, mutations of these genes were not found in this patient by WES, although as mentioned, the main phenotype of the patient in this study includes AVSD and polydactyly.

### 4.1 Two heterozygous mutations are possibly related to the clinical phenotype

Almost 20,000 variants were detected by WES in human genome, by filtering based on allele frequency, function, and genotype‐phenotype relationship, we identified two heterozygous mutations, TCTN3 c.1268G>A and LTBP2 c.2206G>A.

Tectonic‐3 (TCTN3) is part of the tectonic‐like complex, which is required for tissue‐specific ciliogenesis and may regulate ciliary membrane composition. TCTN3 acts as regulators of the sonic hedgehog signalling (Shh) pathway that is essential for cell fate and organ development.^21^

Previous studies demonstrated that variants in TCTN3 lead to Joubert syndrome (JBTS18, OMIM #614815) that involves cardiac and skeletal defects. Patients with JBTS18 have vermis agenesis and the molar tooth sign as well as severe kyphoscoliosis. Other features of JBTS18 include intrauterine growth retardation, oral anomalies, micrognathism, polydactyly and camptodactyly, joint laxity, horseshoe kidney, and ventricular septal defect.^22^

LTBP2 encodes latent‐transforming growth factor beta‐binding protein 2 that plays an integral structural role in elastic‐fibre architectural organization and/or assembly.^23^ LTBP2 with variants can lead to Weill‐Marchesani syndrome 3 (WMS3, OMIM #614819). WMS3 is a rare connective tissue disorder characterized by short stature, brachydactyly, joint stiffness, and eye abnormalities; about 39% patients are accompanied by pulmonary and aortic stenosis.^24^ Nevertheless, the mutations found in both TCTN3 and LTBP2 in this study have not been previously reported and are novel.

Thus, these two genes are related to both congenital heart defects and digital abnormalities and therefore are likely to be associated with phenotypes of the patient in this study.

### 4.2 Functional changes of cardiomyocytes associated with mutations of LTBP2 and TCTN3

During the process of differentiation, we calculated the contraction rate of hPSCs‐CMs with mutation and find that the group hPSCs‐CM‐LTBP2^mu^ was significantly slower than hPSCs‐CM‐WT and the rate increased with further cell culture. This phenomenon indicates that LTBP2 mutation may result in changes of the rhythm development of cardiomyocytes. In contrast, the group hPSCs‐CM‐TCTN3^mu^ showed not only significantly lower rate but also weaker force of contraction. These results suggest that the mutations of LTBP2 and TCTN3 affect early development of the cardiomyocytes regarding the cardiac rhythm and contraction.

### 4.3 Difference of gene expression pattern in hPSCs‐CM‐WT/LTBP2^mu^ or/TCTN3^mu^

For gene expression pattern of persistent up‐regulation, some signal pathways in cardiac development and congenital heart disease were enriched in the group hPSCs‐CM‐LTBP2^mu^ but not in group hPSCs‐CM‐WT, such as HCM,^25^ DCM,^26^ ARVC,^27^ vascular smooth muscle contraction,^28^ and cardiac muscle contraction.^29^ It is possible that changes of these pathways may affect the cardiac development and function. In contrast to the changes of gene expression pattern of persistent up‐regulation in the group hPSCs‐CM‐LTBP2^mu^, there was no persistent up‐regulated expression pattern in group hPSCs‐CM‐ TCTN3^mu^ found in this study.

### 4.4 Translational significance in cardiovascular medicine

The present study demonstrated that in clinically seen CHD associated with polydactyly, mutations of genes other than TBX5 may be involved. Owing to the complexity of the CHD in this study, for personalized diagnosis and treatment in CHD patients associated with polydactyly, WES may be necessary to identify the pathological mutations in the future.

### 4.5 Study limitation

This study has some limitations. Owing to the fact that the patient is an orphan who underwent repair surgery through our charity programme, no family history and data were available.

The present study demonstrates that in complex CHD associated with polydactyly, the mutations of LTBP2 and TCTN3 may be potential pathological cause since these mutations are associated with changes of cellular functions that may affect the development of the heart. Further, this study again demonstrates that TBX5 mutations may not present in complex CHD associated with polydactyly.

In summary, two heterozygous mutations in LTBP2 and TCTN3 without TBX5 mutations were identified in this study. Cellular functional analysis suggested that these two mutations affect contractility (rate and force) of cardiac myocytes and may affect the development of the heart. These findings provide new insights into the pathogenesis of complex CHD associated with polydactyly.

## CONFLICT OF INTEREST

The authors confirm that there are no conflicts of interest.

## AUTHOR CONTRIBUTIONS

Huan‐Xin Chen: Data curation (equal); Formal analysis (equal); Investigation (equal); Methodology (equal); Resources (equal); Validation (equal); Writing‐original draft (equal). Zi‐Yue Yang: Data curation (equal); Formal analysis (equal); Investigation (equal); Methodology (equal); Resources (equal); Validation (equal). Hai‐Tao Hou: Data curation (supporting). Jun Wang: Funding acquisition (supporting); Project administration (equal). Xiu‐Li Wang: Resources (equal). Qin Yang: Data curation (equal); Funding acquisition (supporting); Supervision (equal). Lin Liu: Conceptualization (supporting); Data curation (equal); Supervision (supporting). Guo‐Wei He: Conceptualization (lead); Data curation (equal); Formal analysis (lead); Funding acquisition (lead); Investigation (lead); Methodology (lead); Project administration (lead); Resources (lead); Supervision (lead); Validation (lead); Visualization (lead); Writing‐original draft (lead); Writing‐review & editing (lead).

## Supporting information

Table S1

Table S2

Table S3

Video S1

Video S2

Video S3

Video S4

Supplementary Material

## Data Availability

The data that support the findings of this study are available upon request from the corresponding author.
