# Supplementary material for: Novel mutations of TCTN3/LTBP2 with cellular function changes in congenital heart disease associated with polydactyly
Source: J Cell Mol Med. 2020 Oct 24;24(23):13751–62. doi: 10.1111/jcmm.15950 (PMC7753982; doi:10.1111/jcmm.15950)
Supplement: Supplementary file 1 — Table S1 [file JCMM-24-13751-s001.docx]

**Table S1. Primer Sets Used for Amplification**

|  | F | R |
| --- | --- | --- |
| Tctn3 Genotyping | TTTAGAGCTGGGTGGCCTGT | TCTATGGAGACAAGGCTGGTTTTA |
| Ltbp2 Genotyping | GGCAGCGAGTGTGAGAAATG | CTTTCCCACCTCCACAAGCA |
| Tctn3 template | TCTCATATTTCTGGGCAGTCAG | CCAGCCTAGGTAAGAGTTCAAG |
| Tctn3 sgRNA | CACCGCAATATCTGGATGCAAGCTC | AAACGAGCTTGCATCCAGATATTGC |
| Tctn3 mutation | ATGAAGTGCAGTTTGAAGTGAATGCAATATC | GATATTGCATTCACTTCAAACTGCACTTCAT |
| Tctn3 null-sense mutation | TCTGGATGCAAGCTCAGATAGTTCTTTGCTTCCG | CGGAAGCAAAGAACTATCTGAGCTTGCATCCAGA |
| Ltbp2 template | CATAGCTTCAACAAGCATTACTAAGCATTTGC | CTCGGGTGACAGGAGTCCCACCTGCTCCC |
| Ltbp2 sgRNA | CACCGAGCTCGCGTAGGTGTAGCCG | AAACCGGCTACACCTACGCGAGCTC |
| Ltbp2 mutation | CACCTACGCGAGCTCCAACATCCGCCTGTCCA | TGGACAGGCGGATGTTGGAGCTCGCGTAGGTG |
| Ltbp2 null-sense mutation | GATCTGCCCTGCCGGACACGGCTACACCTACG | CGTAGGTGTAGCCGTGTCCGGCAGGGCAGATC |
|  |  |  |
| Tnnt2 for qPCR | TTCACCAAAGATCTGCTCCTCGCT | TTATTACTGGTGTGGAGTGGGTGTGG |
| Gapdh for qPCR | TCGACAGTCAGCCGCATCTTCTTT | ACCAAATCCGTTGACTCCGACCTT |
| Nanog for qPCR | TTTGGAAGCTGCTGGGGAAG | GATGGGAGGAGGGGAGAGGA |
| Oct4 for qPCR | GACAGGGGGAGGGGAGGAGCTAGG | CTTCCCTCCAACCAGTTGCCCCAAAC |
| Ltbp2 for qPCR | CGGAGACTGCATTGACATAGAC | TAAGCTCACACTCGTTCACATC |
| Tctn3 for qPCR | GCTAGTAGCTGTACCTTGGATTC | CAAGAGTGGCTTCCCAACTAT |
