# Supplementary material for: Novel mutations of TCTN3/LTBP2 with cellular function changes in congenital heart disease associated with polydactyly
Source: J Cell Mol Med. 2020 Oct 24;24(23):13751–62. doi: 10.1111/jcmm.15950 (PMC7753982; doi:10.1111/jcmm.15950)
Supplement: Supplementary file 2 — Table S2 [file JCMM-24-13751-s002.docx]

**Table S2. Information of 137 variants in 123 genes**

| **Gene** | **Transcript** | **Nucleotide change*** | **Amino acid change** | **Function*** | **RS** | **Zygosity** | **Fr.1*** | **Fr.2*** | **Fr.3*** | **Fr.4*** | **PhyloP Vertebrates** | **SIFT** | **Polyphen2 HumDiv** | **Condel Pred*** |
| --- | --- | --- | --- | --- | --- | --- | --- | --- | --- | --- | --- | --- | --- | --- |
| *SDC3* | NM_014654.3 | c.1064G>A | p.R355H \| p.Arg355His | missense | rs201560193 | het-ref | 0.0005 | 0.0017 | . | 0.0007 | 0.206 | 0.25 | 0.991 | neutral |
| *PTCH2* | NM_003738.4 | c.2248G>A | p.A750T \| p.Ala750Thr | missense | . | het-ref | . | . | . | 0.0025 | 0.629 | 0.2 | 0.003 | neutral |
| *CTH* | NM_001902.5 | c.901G>A | p.E301K \| p.Glu301Lys | missense | rs199841412 | het-ref | 0.0005 | 0.0017 | . | 0.0012 | 5.853 | 0.1 | 0.173 | neutral |
| *HMCN1* | NM_031935.2 | c.11915C>T | p.A3972V \| p.Ala3972Val | missense | . | het-ref | . | . | . | 0 | 3.424 | 0.13 | 0.824 | deleterious |
| *DISC1* | NM_001164537.1 | c.983C>A | p.T328N \| p.Thr328Asn | missense | rs55795950 | het-ref | 0.004323 | 0.01 | . | 0.006 | 1.864 | . | . | . |
| *LYST* | NM_000081.2 | c.7997T>A | p.I2666N \| p.Ile2666Asn | missense | . | het-ref | . | . | . | 0.0025 | 3.148 | 0.01 | 0.717 | deleterious |
| *LYST* | NM_000081.2 | c.695G>C | p.G232A \| p.Gly232Ala | missense | . | het-ref | . | . | . | 0.0005 | 0.161 | 0.59 | 0 | neutral |
| *AKR1C4* | NM_001818.3 | c.772C>T | p.R258C \| p.Arg258Cys | missense | . | het-ref | . | . | . | 0 | 1.319 | . | . | . |
| *CUBN* | NM_001081.3 | c.8912C>G | p.S2971C \| p.Ser2971Cys | missense | . | het-ref | . | . | . | 0 | 2.425 | 0.14 | 0.922 | deleterious |
| *RET* | NM_020975.4 | c.2041C>G | p.Q681E \| p.Gln681Glu | missense | . | het-ref | . | . | . | 0.0005 | 5.336 | 0.15 | 0.998 | deleterious |
| *C10orf11* | NM_032024.3 | c.32A>C | p.H11P \| p.His11Pro | missense | . | het-ref | . | . | . | 0.0015 | 2.067 | 0.26 | 0.013 | neutral |
| *TCTN3* | NM_015631.5 | c.1268G>A | p.G423E \| p.Gly423Glu | missense | . | het-ref | . | . | . | 0.0015 | 3.121 | 0.01 | 1 | deleterious |
| *GOT1* | NM_002079.2 | c.1108C>G | p.Q370E \| p.Gln370Glu | missense | rs76850691 | het-ref | 0.002377 | 0.01 | 0.000077 | 0.0086 | 5.899 | 0 | 0.998 | deleterious |
| *FGF8* | NM_033163.3 | c.526G>A | p.E176K \| p.Glu176Lys | missense | rs201979353 | het-ref | 0.0005 | 0.0017 | . | 0.0008 | 3.205 | 0.31 | 0.592 | neutral |
| *USH1C* | NM_153676.3 | c.2551T>G | p.S851A \| p.Ser851Ala | missense | rs200779709 | het-ref | 0.0005 | 0.0017 | 0 | 0.0012 | 0.601 | 0.12 | 0 | neutral |
| *OTOG* | XM_291816.8 | c.2212C>T | p.R738C \| p.Arg738Cys | missense | . | het-ref | . | . | . | . | 2.413 | 0 | 0.999 | deleterious |
| *FGF3* | NM_005247.2 | c.629G>A | p.R210Q \| p.Arg210Gln | missense | rs115452181 | het-ref | 0.002128 | 0.01 | . | 0.0045 | 1.472 | 0.1 | 0.011 | neutral |
| *MAML2* | NM_032427.1 | c.1812_1820delGCAGCAGCA | p.Q619_Q621del \| p.Gln619_Gln621del | cds-del | rs141671766 | hom-alt | 0 | . | . | . | . | . | . | . |
| *DYNC2H1* | NM_001080463.1 | c.5177G>A | p.R1726Q \| p.Arg1726Gln | missense | . | het-ref | . | . | . | . | 5.558 | 0 | 1 | deleterious |
| *CEP164* | NM_014956.4 | c.3931A>C | p.T1311P \| p.Thr1311Pro | missense | . | het-ref | . | . | . | 0 | -0.521 | 0.34 | 0.001 | neutral |
| *KCNA5* | NM_002234.3 | c.236C>T | p.P79L \| p.Pro79Leu | missense | . | het-ref | . | . | . | 0 | 0.985 | 0 | 0.009 | neutral |
| *ATN1* | NM_001007026.1 | c.1508_1509insGCAGCAGCA | p.Q502_H503insQQQ \| p.Gln502_His503insGlnGlnGln | cds-ins | . | het-alt | . | . | . | . | . | . | . | . |
| *ATN1* | NM_001007026.1 | c.1508_1509insGCAGCA | p.Q502_H503insQQ \| p.Gln502_His503insGlnGln | cds-ins | . | het-alt | . | . | . | . | . | . | . | . |
| *DNM1L* | NM_012062.3 | c.511C>A | p.L171I \| p.Leu171Ile | missense | . | het-ref | . | . | . | 0.0007 | 2.929 | 0.06 | 0.014 | neutral |
| *KRT2* | NM_000423.2 | c.311_312insCGGCAGCGGCTTTGGAGG | p.F108_S109insGGGSGF \| p.Gly103_Phe108dup | cds-ins | . | het-ref | . | . | . | . | . | . | . | . |
| *PUS1* | NM_025215.5 | c.1076C>T | p.A359V \| p.Ala359Val | missense | rs142072030 | het-ref | 0.000447 | 0.0017 | 0.000154 | 0.0004 | 0.19 | 0.31 | 0.006 | neutral |
| *ALOX5AP* | NM_001204406.1 | c.116_116+1insGTGT | p.A40Cfs*17 \| p.Ala40Cysfs*17 | frameshift | . | het-ref | . | . | . | . | . | . | . | . |
| *ALOX5AP* | NM_001204406.1 | c.116+1_116+2insTA | . | splice-5 | rs147763360 | het-ref | 0 | . | . | . | . | . | . | . |
| *RFXAP* | NM_000538.3 | c.359G>T | p.G120V \| p.Gly120Val | missense | . | het-ref | . | 0 | . | 0.001 | 2.182 | 0 | 0.999 | deleterious |
| *FREM2* | NM_207361.4 | c.2486C>T | p.P829L \| p.Pro829Leu | missense | . | het-ref | . | . | . | 0 | 6.24 | 0.03 | 1 | deleterious |
| *RB1* | NM_000321.2 | c.2455C>G | p.L819V \| p.Leu819Val | missense | . | het-ref | . | . | 0 | 0.0022 | 0.136 | 0.17 | 0.774 | neutral |
| *EDNRB* | NM_001201397.1 | c.245G>A | p.R82Q \| p.Arg82Gln | missense | rs2070591 | het-ref | 0.009918 | 0.01 | . | 0.0055 | -0.1 | 0 | 0.427 | neutral |
| *SLC10A2* | NM_000452.2 | c.707C>T | p.A236V \| p.Ala236Val | missense | . | het-ref | . | . | . | 0 | 2.706 | . | . | . |
| *CHD8* | NM_001170629.1 | c.7499A>C | p.H2500P \| p.His2500Pro | missense | . | het-ref | . | . | . | . | 2.868 | . | . | . |
| *TGM1* | NM_000359.2 | c.167C>T | p.A56V \| p.Ala56Val | missense | rs147479810 | het-ref | 0.000299 | 0.0017 | 0.000077 | 0.0036 | -0.85 | 0.28 | 0.114 | neutral |
| *DDHD1* | NM_001160148.1 | c.336_337insGGCGGC | p.G112_S113insGG\| p.Gly112_Ser113insGlyGly | cds-ins | rs140904345 | hom-alt | 0 | . | . | . | . | . | . | . |
| *LTBP2* | NM_000428.2 | c.2206G>A | p.D736N \| p.Asp736Asn | missense | rs200629871 | het-ref | 0.0009 | 0.0035 | . | 0.0008 | 3.278 | 0.02 | 0.966 | deleterious |
| *DMXL2* | NM_001174116.1 | c.8191A>G | p.T2731A \| p.Thr2731Ala | missense | . | het-ref | . | . | . | 0 | -0.084 | 0.67 | 0 | neutral |
| *MESP2* | NM_001039958.1 | c.558_581delGGGGCAGGGGCAAGGGCAGGGGCA | p.Q198_G205del \| p.Gln198_Gly205del | cds-del | . | hom-alt | . | . | . | . | . | . | . | . |
| *MEF2A* | NM_005587.2 | c.1262_1267delAGCAGC | p.Q421_Q422del \| p.Gln421_Gln422del | cds-del | rs3138597 | het-alt | 0 | . | . | . | . | . | . | . |
| *MEF2A* | NM_005587.2 | c.1265_1267delAGC | p.Q422del \| p.Gln422del | cds-del | rs3138597 | het-alt | 0 | . | . | . | . | . | . | . |
| *TSC2* | NM_000548.3 | c.3421G>A | p.A1141T \| p.Ala1141Thr | missense | rs45505895 | het-ref | 0 | . | 0.000077 | 0.0029 | 3.133 | 0.33 | 0.001 | neutral |
| *CREBBP* | NM_004380.2 | c.760G>A | p.A254T \| p.Ala254Thr | missense | rs148781922 | het-ref | 0.00022 | . | 0.000231 | 0.0089 | 1.078 | 0.59 | 0.598 | neutral |
| *KARS* | NM_001130089.1 | c.685T>C | p.Y229H \| p.Tyr229His | missense | rs150529876 | het-ref | 0.001337 | 0.01 | 0.000154 | 0.005 | -0.014 | 0.59 | 0 | neutral |
| *WWOX* | NM_016373.2 | c.467G>T | p.R156M \| p.Arg156Met | missense | . | het-ref | . | . | . | . | 4.005 | 0 | 0.999 | deleterious |
| *WWOX* | NM_016373.2 | c.468G>T | p.R156S \| p.Arg156Ser | missense | rs140817689 | het-ref | 0.0018 | 0.01 | 0.000409 | 0.0018 | 2.526 | 0 | 0.998 | deleterious |
| *ZNF469* | NM_001127464.1 | c.946G>A | p.E316K \| p.Glu316Lys | missense | . | het-ref | . | . | . | . | 1.476 | 0.08 | 0.817 | deleterious |
| *ZMYND15* | NM_001267822.1 | c.1543G>A | p.V515M \| p.Val515Met | missense | rs140082251 | het-ref | 0.0027 | 0.01 | . | 0.0031 | 1.415 | 0.06 | 1 | deleterious |
| *RAI1* | NM_030665.3 | c.864_872delGCAGCAGCA | p.Q289_Q291del \| p.Gln289_Gln291del | cds-del | . | het-alt | . | . | . | . | . | . | . | . |
| *RAI1* | NM_030665.3 | c.870_872delGCA | p.Q291del \| p.Gln291del | cds-del | . | het-alt | . | . | . | . | . | . | . | . |
| *B9D1* | NM_001243473.1 | c.715C>A | p.P239T \| p.Pro239Thr | missense | . | het-ref | . | . | . | . | 1.009 | . | . | . |
| *ITGA3* | NM_005501.2 | c.2518G>A | p.G840S \| p.Gly840Ser | missense | rs2301626 | het-ref | 0.00328 | 0.01 | . | 0.005 | 2.98 | 0.08 | 0.026 | neutral |
| *EPX* | NM_000502.4 | c.337A>C | p.N113H \| p.Asn113His | missense | . | het-ref | . | . | . | 0 | 3.22 | 0.05 | 0.999 | deleterious |
| *ACE* | NM_000789.3 | c.1057G>A | p.D353N \| p.Asp353Asn | missense | rs148193919 | het-ref | 0.0018 | 0.0017 | 0.000077 | 0.0025 | 5.56 | 0 | 1 | deleterious |
| *AXIN2* | NM_004655.3 | c.1250C>T | p.A417V \| p.Ala417Val | missense | rs201460658 | het-ref | 0.0032 | 0.01 | . | 0.0047 | 0.29 | 0.28 | 0.013 | neutral |
| *FSCN2* | NM_001077182.2 | c.1106G>A | p.G369E \| p.Gly369Glu | missense | . | het-ref | . | . | . | . | -0.061 | 0.13 | 0.917 | neutral |
| *PIEZO2* | NM_022068.2 | c.1943C>T | p.A648V \| p.Ala648Val | missense | . | het-ref | . | . | . | . | -0.209 | 0.56 | 0.001 | neutral |
| *DSC3* | NM_001941.3 | c.1900C>T | p.R634C \| p.Arg634Cys | missense | . | het-ref | . | . | . | 0 | 2.004 | 0.17 | 0.999 | neutral |
| *DSC2* | NM_024422.3 | c.2287G>A | p.A763T \| p.Ala763Thr | missense | . | het-ref | . | . | . | 0 | -0.199 | 0.57 | 0.006 | neutral |
| *ASXL3* | NM_030632.1 | c.5297A>G | p.Q1766R \| p.Gln1766Arg | missense | . | het-ref | . | . | . | . | 4.012 | 0 | 0.986 | deleterious |
| *MYO5B* | NM_001080467.2 | c.4145C>T | p.T1382M \| p.Thr1382Met | missense | rs145598498 | het-ref | 0.002654 | . | 0.006382 | 0.0027 | 2.263 | 0.68 | 0.295 | neutral |
| *TICAM1* | NM_182919.3 | c.1099_1101dupCCT | p.P367dup \| p.Pro367dup | cds-ins | rs144100421 | het-ref | 0 | . | . | . | . | . | . | . |
| *LONP1* | NM_004793.2 | c.2392G>A | p.G798S \| p.Gly798Ser | missense | rs201378376 | het-ref | 0.0014 | 0.01 | . | 0.0068 | 3.212 | 0.08 | 0.985 | deleterious |
| *PNPLA6* | NM_001166111.1 | c.3458C>T | p.T1153M \| p.Thr1153Met | missense | rs142294971 | het-ref | 0.0003 | 0.0017 | 0.000154 | 0.0005 | 2.624 | 0.23 | 0.963 | neutral |
| *SCN1B* | NM_199037.3 | c.412G>A | p.V138I \| p.Val138Ile | missense | rs72558029 | het-ref | 0.000297 | 0.0017 | 0.000231 | 0.0019 | 2.155 | 0.19 | 0.99 | neutral |
| *NLRP12* | NM_144687.2 | c.1182C>G | p.N394K \| p.Asn394Lys | missense | rs201241894 | het-ref | 0.0009 | 0.0017 | . | 0.0031 | -0.047 | 0.01 | 0.013 | neutral |
| *GP6* | NM_001083899.1 | c.643G>A | p.E215K \| p.Glu215Lys | missense | . | het-ref | . | . | . | . | -0.038 | . | . | . |
| *KLF11* | NM_003597.4 | c.793G>A | p.E265K \| p.Glu265Lys | missense | . | het-ref | . | . | . | 0.0005 | 2.492 | 0.21 | 0.614 | neutral |
| *WDR35* | NM_001006657.1 | c.1058G>C | p.R353P \| p.Arg353Pro | missense | rs76623454 | het-ref | 0.000993 | 0.01 | 0.000154 | 0.0067 | 5.909 | 0.02 | 0.998 | deleterious |
| *GGCX* | NM_000821.5 | c.1378G>A | p.V460I \| p.Val460Ile | missense | rs149078813 | het-ref | 0.0018 | 0.01 | . | 0.0014 | 3.141 | 1 | 0.003 | neutral |
| *SLC5A7* | NM_021815.2 | c.1529A>C | p.K510T \| p.Lys510Thr | missense | rs199693962 | het-ref | 0.0005 | 0.0017 | . | 0.0024 | 3.69 | 0.06 | 0.951 | deleterious |
| *TTN* | NM_001267550.1 | c.74042A>G | p.Q24681R \| p.Gln24681Arg | missense | . | het-ref | . | . | . | . | 3.554 | . | . | . |
| *TTN* | NM_001267550.1 | c.16082A>G | p.K5361R \| p.Lys5361Arg | missense | . | het-ref | . | . | . | . | 5.306 | . | . | . |
| *PGAP1* | NM_024989.3 | c.2467C>G | p.L823V \| p.Leu823Val | missense | rs138036688 | het-ref | 0.0027 | 0.01 | . | 0.0053 | -1.26 | 0.64 | 0 | neutral |
| *GIGYF2* | NM_001103147.1 | c.986T>G | p.L329R \| p.Leu329Arg | missense | rs115367247 | het-ref | 0.004717 | . | . | 0 | 3.337 | 0 | 0.998 | deleterious |
| *KIF1A* | NM_001244008.1 | c.2751_2753delGGA | p.E917del \| p.Glu917del | cds-del | rs10594016 | hom-alt | 0 | . | . | . | . | . | . | . |
| *PROKR2* | NM_144773.2 | c.151G>A | p.A51T \| p.Ala51Thr | missense | rs144994507 | het-ref | 0.002356 | 0.01 | 0.000769 | 0.0096 | 2.487 | 0.17 | 0.068 | neutral |
| *PLK1S1* | NM_018474.4 | c.961T>C | p.S321P \| p.Ser321Pro | missense | rs116937124 | het-ref | 0.002604 | 0.01 | . | 0.0018 | 0.352 | . | . | . |
| *MYLK2* | NM_033118.3 | c.281C>T | p.P94L \| p.Pro94Leu | missense | rs201134349 | het-ref | 0.0005 | 0.0017 | . | 0.0008 | 0.344 | 0.21 | 0.042 | neutral |
| *AHCY* | NM_000687.2 | c.985C>T | p.R329W \| p.Arg329Trp | missense | . | het-ref | . | . | . | 0.0025 | -0.0657 | 0.25 | 0.001 | neutral |
| *COL18A1* | NM_030582.3 | c.1932C>G | p.D644E \| p.Asp644Glu | missense | . | het-ref | . | . | . | . | 2.657 | . | . | . |
| *FTCD* | NM_006657.2 | c.1168A>G | p.T390A \| p.Thr390Ala | missense | rs201828652 | het-ref | 0.0037 | 0.01 | . | 0.0034 | 1.02 | 0.73 | 0.001 | neutral |
| *PCNT* | NM_006031.5 | c.1784C>T | p.A595V \| p.Ala595Val | missense | rs143028464 | het-ref | 0.00208 | . | 0.001999 | 0.0009 | -0.321 | 0.31 | 0.001 | neutral |
| *IL17RA* | NM_014339.5 | c.7delG | p.A3Pfs*46 \| p.Ala3Profs*46 | frameshift | . | het-ref | . | . | . | 0 | 0.881 | . | . | . |
| *IL17RA* | NM_014339.5 | c.8C>T | p.A3V \| p.Ala3Val | missense | . | het-ref | . | . | . | 0 | 0.041 | . | . | . |
| *TUBA8* | NM_018943.2 | c.640C>T | p.R214C \| p.Arg214Cys | missense | . | het-ref | . | . | . | 0.0017 | 4.211 | 0.1 | 0.965 | neutral |
| *CHCHD10* | NM_213720.1 | c.268A>C | p.T90P \| p.Thr90Pro | missense | . | het-ref | . | . | . | 0 | -0.576 | 0.38 | 0.001 | neutral |
| *LARGE* | NM_004737.4 | c.1556G>A | p.G519D \| p.Gly519Asp | missense | . | het-ref | . | . | . | 0 | 3.964 | 0.61 | 0.018 | neutral |
| *MYH9* | NM_002473.4 | c.3913G>A | p.A1305T \| p.Ala1305Thr | missense | . | het-ref | . | . | . | 0 | -0.587 | 0.47 | 0.001 | neutral |
| *MKL1* | NM_020831.3 | c.1705G>C | p.A569P \| p.Ala569Pro | missense | . | het-ref | . | 0 | 0 | 0 | 0.394 | 0.15 | 0.493 | neutral |
| *MKL1* | NM_020831.3 | c.1699G>C | p.A567P \| p.Ala567Pro | missense | . | het-ref | . | . | 0 | 0 | 0.17 | 0.32 | 0.904 | neutral |
| *SBF1* | NM_002972.2 | c.2991G>C | p.E997D \| p.Glu997Asp | missense | . | het-ref | . | . | . | . | 1.533 | 0.17 | 0.998 | deleterious |
| *FYCO1* | NM_024513.3 | c.1996G>A | p.E666K \| p.Glu666Lys | missense | . | het-ref | . | . | 0.000077 | 0 | 1.714 | 0.12 | 0.035 | neutral |
| *TMIE* | NM_147196.2 | c.391_393delAAG | p.K131del \| p.Lys131del | cds-del | rs10578999 | hom-alt | 0 | . | . | . | . | . | . | . |
| *NBEAL2* | NM_015175.2 | c.631G>A | p.A211T \| p.Ala211Thr | missense | . | het-ref | . | . | . | . | 1.404 | 0.42 | 0.059 | neutral |
| *HYAL1* | NM_153281.1 | c.845C>T | p.P282L \| p.Pro282Leu | missense | . | het-ref | . | . | . | 0 | 1.429 | 0 | 0.985 | deleterious |
| *CISH* | NM_013324.5 | c.77G>C | p.R26P \| p.Arg26Pro | missense | . | het-ref | . | . | . | 0 | 0.778 | 0.33 | 0.013 | neutral |
| *HRG* | NM_000412.2 | c.1256C>T | p.P419L \| p.Pro419Leu | missense | . | het-ref | . | . | . | 0 | 1.578 | 0.06 | 0.994 | deleterious |
| *FRAS1* | NM_025074.6 | c.6470T>A | p.V2157E \| p.Val2157Glu | missense | . | het-ref | . | . | . | . | 4.997 | 0 | 0.002 | neutral |
| *ANK2* | NM_001148.4 | c.8240G>A | p.R2747H \| p.Arg2747His | missense | rs142137451 | het-ref | 0.000743 | 0.01 | 0.000154 | 0.0039 | 0.362 | 0.04 | 0.02 | neutral |
| *FAT4* | NM_024582.4 | c.11492A>G | p.E3831G \| p.Glu3831Gly | missense | . | het-ref | . | . | . | 0.0005 | 5.019 | 0.01 | 0.996 | deleterious |
| *PLK4* | NM_014264.4 | c.2483C>G | p.T828R \| p.Thr828Arg | missense | . | het-ref | . | 0 | 0 | 0 | 0.013 | 0.56 | 0 | neutral |
| *TENM3* | NM_001080477.1 | c.4580A>G | p.N1527S \| p.Asn1527Ser | missense | rs202067365 | het-ref | 0 | . | 0.000248 | . | 4.965 | 0.54 | 0.129 | neutral |
| *MSH3* | NM_002439.4 | c.181_189dupGCAGCGCCC | p.A61_P63dup \| p.Ala61_Pro63dup | cds-ins | . | het-ref | . | 0 | . | . | . | . | . | . |
| *MCC* | NM_001085377.1 | c.61_63dupGGC | p.G21dup \| p.Gly21dup | cds-ins | . | hom-alt | . | . | . | . | . | . | . | . |
| *DIAPH1* | NM_005219.4 | c.1853_1854insTCCTCC | p.P620_L621insPP\| p.Pro620_Leu621insProPro | cds-ins | . | het-ref | . | . | . | . | . | . | . | . |
| *HAVCR1* | NM_012206.2 | c.473_487delTGACGACTGTTCCAA | p.M158_P162del \| p.Met158_Pro162del | cds-del | . | het-alt | . | . | . | . | . | . | . | . |
| *HAVCR1* | NM_012206.2 | c.476_477insAAC | p.T160dup \| p.Thr160dup | cds-ins | . | het-alt | . | . | . | . | . | . | . | . |
| *DSP* | NM_004415.2 | c.449G>A | p.R150Q \| p.Arg150Gln | missense | . | het-ref | . | . | . | 0 | 4.417 | 0.36 | 0.997 | neutral |
| *DSP* | NM_004415.2 | c.3923G>A | p.R1308Q \| p.Arg1308Gln | missense | rs184154918 | het-ref | 0.0027 | 0.01 | 0.000077 | 0.0062 | 1.432 | 0.09 | 0.827 | neutral |
| *ATXN1* | NM_000332.3 | c.624_626dupGCA | p.Q208dup \| p.Gln208dup | cds-ins | . | het-ref | . | . | . | . | . | . | . | . |
| *C2* | NM_000063.4 | c.325G>A | p.V109M \| p.Val109Met | missense | . | het-ref | . | . | . | 0.0007 | 2.498 | 0.08 | 1 | deleterious |
| *HLA-DQB1* | NM_001243961.1 | c.362G>C | p.G121A \| p.Gly121Ala | missense | . | het-ref | . | . | . | . | -1.219 | 0.08 | 0 | neutral |
| *HLA-DQB1* | NM_001243961.1 | c.361G>A | p.G121R \| p.Gly121Arg | missense | . | het-ref | . | . | . | . | -3.278 | 0.04 | 0.003 | neutral |
| *HLA-DQB1* | NM_001243961.1 | c.353C>A | p.A118E \| p.Ala118Glu | missense | . | het-ref | . | 0 | 0 | 0 | -0.901 | 0.3 | 0.002 | neutral |
| *RSPH9* | NM_001193341.1 | c.912_913delCA | p.N304Kfs*? \| p.Asn304Lysfs*? | frameshift | . | het-ref | . | . | . | . | . | . | . | . |
| *TBP* | NM_003194.4 | c.273_281delGCAGCAGCA | p.Q93_Q95del \| p.Gln93_Gln95del | cds-del | . | het-ref | . | . | . | . | . | . | . | . |
| *CARD11* | NM_032415.4 | c.1595C>T | p.T532M \| p.Thr532Met | missense | rs201780608 | het-ref | 0 | . | 0.000077 | 0.0022 | -1.192 | 0.13 | 0.003 | neutral |
| *TWIST1* | NM_000474.3 | c.259_276delGCGGGCGGCGGCGGCGGC | p.A87_G92del \| p.Ala87_Gly92del | cds-del | . | het-ref | . | . | . | . | . | . | . | . |
| *DNAH11* | NM_003777.3 | c.13260A>C | p.K4420N \| p.Lys4420Asn | missense | . | het-ref | . | . | . | . | 0.787 | 0 | 0.999 | deleterious |
| *POU6F2* | NM_007252.3 | c.1333C>T | p.R445W \| p.Arg445Trp | missense | . | het-ref | . | . | . | 0.0025 | 4.147 | . | . | . |
| *AUTS2* | NM_015570.2 | c.385C>G | p.L129V \| p.Leu129Val | missense | rs145480547 | het-ref | 0.000891 | 0.01 | 0.000692 | 0.0043 | 1.892 | 0.2 | 0.624 | neutral |
| *CALCR* | NM_001164737.1 | c.736G>A | p.V246M \| p.Val246Met | missense | . | het-ref | . | . | . | 0 | 3.539 | 0.01 | 1 | deleterious |
| *DNAJB6* | NM_058246.3 | c.700G>A | p.D234N \| p.Asp234Asn | missense | . | het-ref | . | . | . | 0 | 1.776 | . | . | . |
| *RP1L1* | NM_178857.5 | c.3971A>G | p.E1324G \| p.Glu1324Gly | missense | . | het-ref | . | . | . | . | 0.069 | . | . | . |
| *RP1L1* | NM_178857.5 | c.2435G>A | p.R812Q \| p.Arg812Gln | missense | . | het-ref | . | . | . | . | -0.027 | 0.43 | 0.051 | neutral |
| *LZTS1* | NM_021020.2 | c.786C>G | p.S262R \| p.Ser262Arg | missense | . | het-ref | . | . | . | 0.0014 | 1.517 | 0.01 | 0.004 | neutral |
| *SFTPC* | NM_003018.3 | c.68G>A | p.R23Q \| p.Arg23Gln | missense | rs75902455 | het-ref | 0.0014 | 0.01 | . | 0.0014 | 0.709 | 0.29 | 0.281 | neutral |
| *CHD7* | NM_017780.3 | c.6571G>A | p.E2191K \| p.Glu2191Lys | missense | . | het-ref | . | . | . | . | 2.209 | 0.57 | 0.161 | neutral |
| *ZFHX4* | NM_024721.4 | c.6964C>T | p.P2322S \| p.Pro2322Ser | missense | . | het-ref | . | . | . | . | 5.639 | . | . | . |
| *ASPN* | NM_017680.4 | c.150_152delTGA | p.D50del \| p.Asp50del | cds-del | . | hom-alt | . | . | . | . | . | . | . | . |
| *PTCH1* | NM_000264.3 | c.2678G>A | p.R893H \| p.Arg893His | missense | rs138154222 | het-ref | 0.0037 | 0.01 | . | 0.0067 | 2.124 | 0.12 | 0.996 | deleterious |
| *SLC27A4* | NM_005094.3 | c.407G>A | p.R136H \| p.Arg136His | missense | rs148684713 | het-ref | 0.0009 | 0.0035 | . | 0.0045 | 3.821 | 0.04 | 1 | deleterious |
| *LHX3* | NM_014564.3 | c.979G>A | p.A327T \| p.Ala327Thr | missense | rs201356862 | hom-alt | 0.0023 | 0.01 | . | 0.0055 | 2.869 | 0.56 | 0.564 | neutral |
| *SLC34A3* | NM_001177317.1 | c.751G>A | p.V251M \| p.Val251Met | missense | . | het-ref | . | . | . | 0 | 0.882 | . | . | . |
| *MBTPS2* | NM_015884.3 | c.1093G>A | p.V365I \| p.Val365Ile | missense | . | het-ref | . | . | . | 0 | 0.068 | 0.62 | 0.001 | neutral |
| *SHROOM4* | NM_020717.3 | c.3346C>T | p.R1116C \| p.Arg1116Cys | missense | rs200144471 | het-ref | 0.001206 | 0.0047 | . | 0.0023 | 0.983 | 0 | 0.993 | deleterious |
|  |  |  |  |  |  |  |  |  |  |  |  |  |  |  |
| * Nucleotide change: | |  |  |  |  |  |  |  |  |  |  |  |  |  |
| c.1670C>T: the nucleotide C is substituted by T at position 1670 of cDNA. | | | | | | |  |  |  |  |  |  |  |  |
| c.1128+5G>A: the nucleotide G is substituted by A at the position located 5 bases downstream of position 1128 of cDNA. | | | | | | | | | | | |  |  |  |
| * Function (from genetic home reference): | | | |  |  |  |  |  |  |  |  |  |  |  |
| Missense: A single base pair substitution that results in the translation of a different amino acid at that position. | | | | | | | | | | | |  |  |  |
| Splice: A mutation that alters or abolishes the specific sequence denoting the site at which the splicing of an intron takes place. | | | | | | | | | | | | | |  |
| Nonsense: A single base pair substitution that prematurely codes for a stop in amino acid translation (stop codon). | | | | | | | | | | | |  |  |  |
| Frameshift: An insertion or deletion involving a number of base pairs that is not a multiple of three and consequently disrupts the triplet reading frame, usually leading to the creation of a premature | | | | | | | | | | | | | | |
| termination (stop) codon and resulting in a truncated protein product. | | | | | |  |  |  |  |  |  |  |  |  |
| cds-ins: An insertion involving a number of base pairs that is a multiple of three and consequently do not disrupt the triplet reading frame. | | | | | | | | | | | | | | |
| cds-del: An deletion involving a number of base pairs that is a multiple of three and consequently do not disrupt the triplet reading frame. | | | | | | | | | | | | | | |
| * Fr.1: allele frequency in dbSNP. | | |  |  |  |  |  |  |  |  |  |  |  |  |
| * Fr.2: allele frequency in 1000Genome (Asian population) | | | | |  |  |  |  |  |  |  |  |  |  |
| * Fr.3: allele frequency in ESP6500. | | | |  |  |  |  |  |  |  |  |  |  |  |
| * Fr.4: allele frequency in HGVD (BGI). | | | |  |  |  |  |  |  |  |  |  |  |  |
| * PhyloP Vertebrates: the conservative score in vertebrates by PhyloP program (the site with score >3 is considered as high conservative). | | | | | | | | | | | | | |  |
| * SIFT：prediction scores of variations range from 0 (damaging) to 1 (benign). | | | | | | |  |  |  |  |  |  |  |  |
| * Polyphen2HumDiv：prediction scores of variations range from 0 (benign) to 1(damaging). | | | | | | | |  |  |  |  |  |  |  |
| * Condel prediction：the prediction result is either deleterious (damaging) or neutral (tolerant) | | | | | | | | |  |  |  |  |  |  |
