# Supplementary material for: Novel mutations of TCTN3/LTBP2 with cellular function changes in congenital heart disease associated with polydactyly
Source: J Cell Mol Med. 2020 Oct 24;24(23):13751–62. doi: 10.1111/jcmm.15950 (PMC7753982; doi:10.1111/jcmm.15950)
Supplement: Supplementary file 3 — Table S3 [file JCMM-24-13751-s003.docx]

**Table S3. KEGG pathway enrichment analysis of continuous rise pattern in hPSC-CMs-WT and hPSC-CMs-LTBP2mu**

| **Pathway ID** | **Pathway** | **profile7 (370)** | **All (6948)** | **Pvalue** | **Qvalue** |
| --- | --- | --- | --- | --- | --- |
| ko05410 | Hypertrophic cardiomyopathy (HCM) | 18 | 84 | 2.93E-07 | 7.65E-05 |
| ko05414 | Dilated cardiomyopathy (DCM) | 16 | 88 | 1.29E-05 | 1.68E-03 |
| ko04510 | Focal adhesion | 25 | 207 | 9.41E-05 | 6.84E-03 |
| ko05412 | Arrhythmogenic right ventricular cardiomyopathy (ARVC) | 13 | 73 | 0.0001048 | 6.84E-03 |
| ko04210 | Apoptosis | 13 | 90 | 0.0008779 | 4.22E-02 |
| ko05200 | Pathways in cancer | 36 | 395 | 0.000971 | 4.22E-02 |
| ko05205 | Proteoglycans in cancer | 21 | 199 | 0.0019913 | 6.30E-02 |
| ko04921 | Oxytocin signaling pathway | 18 | 161 | 0.0021728 | 6.30E-02 |
| ko05211 | Renal cell carcinoma | 10 | 66 | 0.0023627 | 6.30E-02 |
| ko04810 | Regulation of actin cytoskeleton | 22 | 217 | 0.0026114 | 6.30E-02 |
| ko04261 | Adrenergic signaling in cardiomyocytes | 17 | 151 | 0.0026538 | 6.30E-02 |
| ko04270 | Vascular smooth muscle contraction | 15 | 131 | 0.003949 | 8.59E-02 |
| ko04064 | NF-kappa B signaling pathway | 12 | 97 | 0.0051377 | 1.03E-01 |
| ko04024 | cAMP signaling pathway | 20 | 203 | 0.005553 | 1.04E-01 |
| ko04260 | Cardiac muscle contraction | 10 | 79 | 0.008745 | 1.49E-01 |
| ko00534 | Glycosaminoglycan biosynthesis - heparan sulfate / heparin | 5 | 25 | 0.009153 | 1.49E-01 |
| ko04146 | Peroxisome | 10 | 82 | 0.0112814 | 1.73E-01 |
| ko05218 | Melanoma | 9 | 72 | 0.0136063 | 1.87E-01 |
| ko04320 | Dorso-ventral axis formation | 5 | 28 | 0.0148585 | 1.87E-01 |
| ko04713 | Circadian entrainment | 11 | 99 | 0.0157065 | 1.87E-01 |
| ko04020 | Calcium signaling pathway | 17 | 182 | 0.016653 | 1.87E-01 |
| ko04668 | TNF signaling pathway | 12 | 113 | 0.0166557 | 1.87E-01 |
| ko05133 | Pertussis | 9 | 75 | 0.0174722 | 1.87E-01 |
| ko04012 | ErbB signaling pathway | 10 | 88 | 0.0179791 | 1.87E-01 |
| ko04080 | Neuroactive ligand-receptor interaction | 25 | 302 | 0.01807 | 1.87E-01 |
| ko04933 | AGE-RAGE signaling pathway in diabetic complications | 11 | 102 | 0.019278 | 1.87E-01 |
| ko05150 | Staphylococcus aureus infection | 7 | 52 | 0.0193705 | 1.87E-01 |
| ko04022 | cGMP - PKG signaling pathway | 16 | 174 | 0.02265 | 2.08E-01 |
| ko05214 | Glioma | 8 | 66 | 0.0230769 | 2.08E-01 |
| ko05140 | Leishmaniasis | 8 | 69 | 0.0292998 | 2.50E-01 |
| ko02010 | ABC transporters | 6 | 45 | 0.0307678 | 2.50E-01 |
| ko04614 | Renin-angiotensin system | 4 | 23 | 0.0313568 | 2.50E-01 |
| ko04610 | Complement and coagulation cascades | 8 | 70 | 0.0316084 | 2.50E-01 |
| ko04640 | Hematopoietic cell lineage | 9 | 85 | 0.0361201 | 2.77E-01 |
| ko05144 | Malaria | 6 | 48 | 0.0406225 | 2.98E-01 |
| ko05222 | Small cell lung cancer | 9 | 87 | 0.0410513 | 2.98E-01 |
| ko04014 | Ras signaling pathway | 19 | 234 | 0.0430265 | 3.00E-01 |
| ko00330 | Arginine and proline metabolism | 6 | 49 | 0.0443032 | 3.00E-01 |
| ko05145 | Toxoplasmosis | 11 | 117 | 0.0465516 | 3.00E-01 |
| ko04723 | Retrograde endocannabinoid signaling | 10 | 103 | 0.0468262 | 3.00E-01 |
| ko05161 | Hepatitis B | 13 | 147 | 0.0490307 | 3.00E-01 |
| ko05215 | Prostate cancer | 9 | 90 | 0.0492702 | 3.00E-01 |
| ko05142 | Chagas disease (American trypanosomiasis) | 10 | 104 | 0.0494703 | 3.00E-01 |
| ko04670 | Leukocyte transendothelial migration | 11 | 120 | 0.0541662 | 3.21E-01 |
| ko00450 | Selenocompound metabolism | 3 | 17 | 0.0584502 | 3.39E-01 |
| ko05031 | Amphetamine addiction | 7 | 67 | 0.064339 | 3.65E-01 |
| ko04066 | HIF-1 signaling pathway | 10 | 111 | 0.0707818 | 3.82E-01 |
| ko04010 | MAPK signaling pathway | 20 | 265 | 0.0715838 | 3.82E-01 |
| ko04360 | Axon guidance | 11 | 126 | 0.0717924 | 3.82E-01 |
| ko05416 | Viral myocarditis | 6 | 56 | 0.0757913 | 3.91E-01 |
| ko00532 | Glycosaminoglycan biosynthesis - chondroitin sulfate / dermatan sulfate | 3 | 19 | 0.0770792 | 3.91E-01 |
| ko05223 | Non-small cell lung cancer | 6 | 57 | 0.0811121 | 3.91E-01 |
| ko04350 | TGF-beta signaling pathway | 8 | 85 | 0.0818455 | 3.91E-01 |
| ko04115 | p53 signaling pathway | 7 | 71 | 0.0824245 | 3.91E-01 |
| ko05146 | Amoebiasis | 9 | 100 | 0.0841751 | 3.91E-01 |
| ko04726 | Serotonergic synapse | 10 | 115 | 0.0852242 | 3.91E-01 |
| ko04060 | Cytokine-cytokine receptor interaction | 20 | 271 | 0.0853817 | 3.91E-01 |
| ko05231 | Choline metabolism in cancer | 9 | 101 | 0.0883177 | 3.94E-01 |
| ko04728 | Dopaminergic synapse | 11 | 131 | 0.0889994 | 3.94E-01 |
| ko05206 | MicroRNAs in cancer | 12 | 148 | 0.0953442 | 4.15E-01 |
| ko04130 | SNARE interactions in vesicular transport | 4 | 34 | 0.1045661 | 4.47E-01 |
| ko04974 | Protein digestion and absorption | 8 | 91 | 0.1105644 | 4.59E-01 |
| ko04370 | VEGF signaling pathway | 6 | 62 | 0.1107279 | 4.59E-01 |
| ko04068 | FoxO signaling pathway | 11 | 139 | 0.1213476 | 4.95E-01 |
| ko04015 | Rap1 signaling pathway | 16 | 220 | 0.1260824 | 5.01E-01 |
| ko04912 | GnRH signaling pathway | 8 | 94 | 0.1267464 | 5.01E-01 |
| ko00590 | Arachidonic acid metabolism | 6 | 65 | 0.1308094 | 5.05E-01 |
| ko04540 | Gap junction | 8 | 95 | 0.1324012 | 5.05E-01 |
| ko04071 | Sphingolipid signaling pathway | 10 | 126 | 0.1334999 | 5.05E-01 |
| ko05033 | Nicotine addiction | 4 | 38 | 0.1416537 | 5.28E-01 |
| ko05212 | Pancreatic cancer | 6 | 67 | 0.1450993 | 5.33E-01 |
| ko04978 | Mineral absorption | 5 | 53 | 0.1499185 | 5.36E-01 |
| ko05213 | Endometrial cancer | 5 | 53 | 0.1499185 | 5.36E-01 |
| ko04151 | PI3K-Akt signaling pathway | 23 | 345 | 0.1548255 | 5.46E-01 |
| ko00380 | Tryptophan metabolism | 4 | 40 | 0.1618983 | 5.54E-01 |
| ko04072 | Phospholipase D signaling pathway | 11 | 148 | 0.1646019 | 5.54E-01 |
| ko05134 | Legionellosis | 5 | 55 | 0.1672886 | 5.54E-01 |
| ko03320 | PPAR signaling pathway | 6 | 70 | 0.1677946 | 5.54E-01 |
| ko04720 | Long-term potentiation | 6 | 70 | 0.1677946 | 5.54E-01 |
| ko05219 | Bladder cancer | 4 | 41 | 0.1723943 | 5.57E-01 |
| ko05323 | Rheumatoid arthritis | 7 | 86 | 0.1728839 | 5.57E-01 |
| ko04750 | Inflammatory mediator regulation of TRP channels | 8 | 102 | 0.1754243 | 5.58E-01 |
| ko04727 | GABAergic synapse | 7 | 87 | 0.1800602 | 5.59E-01 |
| ko04911 | Insulin secretion | 7 | 87 | 0.1800602 | 5.59E-01 |
| ko00760 | Nicotinate and nicotinamide metabolism | 3 | 28 | 0.1850139 | 5.68E-01 |
| ko05220 | Chronic myeloid leukemia | 6 | 73 | 0.1918745 | 5.82E-01 |
| ko00730 | Thiamine metabolism | 1 | 4 | 0.1966309 | 5.90E-01 |
| ko04390 | Hippo signaling pathway | 11 | 155 | 0.2028391 | 6.02E-01 |
| ko04611 | Platelet activation | 9 | 123 | 0.2079178 | 6.08E-01 |
| ko04620 | Toll-like receptor signaling pathway | 8 | 107 | 0.2094991 | 6.08E-01 |
| ko05152 | Tuberculosis | 12 | 174 | 0.2162241 | 6.11E-01 |
| ko04971 | Gastric acid secretion | 6 | 76 | 0.2171811 | 6.11E-01 |
| ko04530 | Tight junction | 10 | 142 | 0.2242562 | 6.11E-01 |
| ko00512 | Mucin type O-glycan biosynthesis | 3 | 31 | 0.2270057 | 6.11E-01 |
| ko00514 | Other types of O-glycan biosynthesis | 3 | 31 | 0.2270057 | 6.11E-01 |
| ko00910 | Nitrogen metabolism | 2 | 17 | 0.2283248 | 6.11E-01 |
| ko04514 | Cell adhesion molecules (CAMs) | 10 | 144 | 0.2370389 | 6.11E-01 |
| ko04630 | Jak-STAT signaling pathway | 11 | 161 | 0.2383959 | 6.11E-01 |
| ko00232 | Caffeine metabolism | 1 | 5 | 0.2394371 | 6.11E-01 |
| ko00750 | Vitamin B6 metabolism | 1 | 5 | 0.2394371 | 6.11E-01 |
| ko04973 | Carbohydrate digestion and absorption | 4 | 47 | 0.2396184 | 6.11E-01 |
| ko00410 | beta-Alanine metabolism | 3 | 32 | 0.2413918 | 6.11E-01 |
| ko04150 | mTOR signaling pathway | 5 | 63 | 0.2435266 | 6.11E-01 |
| ko05321 | Inflammatiory bowel disease (IBD) | 5 | 63 | 0.2435266 | 6.11E-01 |
| ko05143 | African trypanosomiasis | 3 | 34 | 0.2705701 | 6.67E-01 |
| ko04512 | ECM-receptor interaction | 6 | 82 | 0.2707879 | 6.67E-01 |
| ko00510 | N-Glycan biosynthesis | 4 | 50 | 0.2752542 | 6.71E-01 |
| ko04961 | Endocrine and other factor-regulated calcium reabsorption | 4 | 52 | 0.2994794 | 7.24E-01 |
| ko04922 | Glucagon signaling pathway | 7 | 104 | 0.3172957 | 7.60E-01 |
| ko04622 | RIG-I-like receptor signaling pathway | 5 | 71 | 0.3269426 | 7.76E-01 |
| ko04722 | Neurotrophin signaling pathway | 8 | 124 | 0.3401321 | 8.00E-01 |
| ko05032 | Morphine addiction | 6 | 90 | 0.3463684 | 8.07E-01 |
| ko05221 | Acute myeloid leukemia | 4 | 57 | 0.3608466 | 8.33E-01 |
| ko04975 | Fat digestion and absorption | 3 | 42 | 0.3889323 | 8.90E-01 |
| ko00260 | Glycine, serine and threonine metabolism | 3 | 43 | 0.403548 | 9.04E-01 |
| ko04666 | Fc gamma R-mediated phagocytosis | 6 | 96 | 0.4043042 | 9.04E-01 |
| ko04725 | Cholinergic synapse | 7 | 114 | 0.4053662 | 9.04E-01 |
| ko05100 | Bacterial invasion of epithelial cells | 5 | 79 | 0.4127709 | 9.13E-01 |
| ko00071 | Fatty acid degradation | 3 | 44 | 0.418062 | 9.17E-01 |
| ko05210 | Colorectal cancer | 4 | 63 | 0.4341853 | 9.42E-01 |
| ko04972 | Pancreatic secretion | 6 | 100 | 0.4427966 | 9.42E-01 |
| ko00563 | Glycosylphosphatidylinositol(GPI)-anchor biosynthesis | 2 | 28 | 0.4439898 | 9.42E-01 |
| ko00650 | Butanoate metabolism | 2 | 28 | 0.4439898 | 9.42E-01 |
| ko04122 | Sulfur relay system | 1 | 11 | 0.4525038 | 9.44E-01 |
| ko04925 | Aldosterone synthesis and secretion | 5 | 83 | 0.4552606 | 9.44E-01 |
| ko04924 | Renin secretion | 4 | 65 | 0.4581756 | 9.44E-01 |
| ko04120 | Ubiquitin mediated proteolysis | 9 | 157 | 0.4595154 | 9.44E-01 |
| ko04721 | Synaptic vesicle cycle | 4 | 66 | 0.4700431 | 9.48E-01 |
| ko05169 | Epstein-Barr virus infection | 11 | 196 | 0.4737023 | 9.48E-01 |
| ko01212 | Fatty acid metabolism | 3 | 48 | 0.4748092 | 9.48E-01 |
| ko05030 | Cocaine addiction | 3 | 49 | 0.4886055 | 9.48E-01 |
| ko04710 | Circadian rhythm | 2 | 31 | 0.4974099 | 9.48E-01 |
| ko04340 | Hedgehog signaling pathway | 3 | 50 | 0.5022236 | 9.48E-01 |
| ko04672 | Intestinal immune network for IgA production | 3 | 50 | 0.5022236 | 9.48E-01 |
| ko04913 | Ovarian Steroidogenesis | 3 | 50 | 0.5022236 | 9.48E-01 |
| ko04910 | Insulin signaling pathway | 8 | 144 | 0.5034793 | 9.48E-01 |
| ko05230 | Central carbon metabolism in cancer | 4 | 69 | 0.505039 | 9.48E-01 |
| ko04550 | Signaling pathways regulating pluripotency of stem cells | 8 | 145 | 0.5114461 | 9.48E-01 |
| ko05168 | Herpes simplex infection | 10 | 183 | 0.5136501 | 9.48E-01 |
| ko00480 | Glutathione metabolism | 3 | 51 | 0.5156545 | 9.48E-01 |
| ko00600 | Sphingolipid metabolism | 3 | 51 | 0.5156545 | 9.48E-01 |
| ko05014 | Amyotrophic lateral sclerosis (ALS) | 3 | 51 | 0.5156545 | 9.48E-01 |
| ko00310 | Lysine degradation | 3 | 52 | 0.5288897 | 9.58E-01 |
| ko00983 | Drug metabolism - other enzymes | 3 | 52 | 0.5288897 | 9.58E-01 |
| ko00790 | Folate biosynthesis | 1 | 14 | 0.5355313 | 9.58E-01 |
| ko04931 | Insulin resistance | 6 | 110 | 0.5361669 | 9.58E-01 |
| ko05217 | Basal cell carcinoma | 3 | 54 | 0.554744 | 9.58E-01 |
| ko00604 | Glycosphingolipid biosynthesis - ganglio series | 1 | 15 | 0.5603155 | 9.58E-01 |
| ko04662 | B cell receptor signaling pathway | 4 | 74 | 0.5609421 | 9.58E-01 |
| ko04917 | Prolactin signaling pathway | 4 | 74 | 0.5609421 | 9.58E-01 |
| ko05160 | Hepatitis C | 7 | 133 | 0.5684071 | 9.58E-01 |
| ko04724 | Glutamatergic synapse | 6 | 115 | 0.5803313 | 9.58E-01 |
| ko00562 | Inositol phosphate metabolism | 4 | 76 | 0.5823267 | 9.58E-01 |
| ko00533 | Glycosaminoglycan biosynthesis - keratan sulfate | 1 | 16 | 0.5837805 | 9.58E-01 |
| ko04923 | Regulation of lipolysis in adipocyte | 3 | 57 | 0.591897 | 9.58E-01 |
| ko05162 | Measles | 7 | 136 | 0.5923715 | 9.58E-01 |
| ko05204 | Chemical carcinogenesis | 4 | 77 | 0.5927924 | 9.58E-01 |
| ko05332 | Graft-versus-host disease | 2 | 37 | 0.5938949 | 9.58E-01 |
| ko01210 | 2-Oxocarboxylic acid metabolism | 1 | 17 | 0.6059965 | 9.58E-01 |
| ko00980 | Metabolism of xenobiotics by cytochrome P450 | 4 | 79 | 0.6132532 | 9.58E-01 |
| ko04621 | NOD-like receptor signaling pathway | 3 | 59 | 0.615528 | 9.58E-01 |
| ko00120 | Primary bile acid biosynthesis | 1 | 18 | 0.6270297 | 9.58E-01 |
| ko00360 | Phenylalanine metabolism | 1 | 18 | 0.6270297 | 9.58E-01 |
| ko04940 | Type I diabetes mellitus | 2 | 40 | 0.6366945 | 9.58E-01 |
| ko04730 | Long-term depression | 3 | 61 | 0.638221 | 9.58E-01 |
| ko00240 | Pyrimidine metabolism | 5 | 102 | 0.6396454 | 9.58E-01 |
| ko00511 | Other glycan degradation | 1 | 19 | 0.646943 | 9.58E-01 |
| ko04960 | Aldosterone-regulated sodium reabsorption | 2 | 41 | 0.6501541 | 9.58E-01 |
| ko04070 | Phosphatidylinositol signaling system | 5 | 104 | 0.6566889 | 9.58E-01 |
| ko04916 | Melanogenesis | 5 | 104 | 0.6566889 | 9.58E-01 |
| ko05203 | Viral carcinogenesis | 10 | 205 | 0.6593484 | 9.58E-01 |
| ko04623 | Cytosolic DNA-sensing pathway | 3 | 63 | 0.6599625 | 9.58E-01 |
| ko00100 | Steroid biosynthesis | 1 | 20 | 0.6657958 | 9.58E-01 |
| ko04919 | Thyroid hormone signaling pathway | 6 | 126 | 0.669335 | 9.58E-01 |
| ko04213 | Longevity regulating pathway - multiple species | 3 | 64 | 0.6704745 | 9.58E-01 |
| ko05010 | Alzheimer's disease | 8 | 167 | 0.6724258 | 9.58E-01 |
| ko01100 | Metabolic pathways | 2 | 43 | 0.6758819 | 9.58E-01 |
| ko04114 | Oocyte meiosis | 6 | 127 | 0.6768057 | 9.58E-01 |
| ko04062 | Chemokine signaling pathway | 9 | 189 | 0.6838798 | 9.58E-01 |
| ko00565 | Ether lipid metabolism | 2 | 44 | 0.6881591 | 9.58E-01 |
| ko04141 | Protein processing in endoplasmic reticulum | 8 | 171 | 0.6979887 | 9.58E-01 |
| ko04932 | Non-alcoholic fatty liver disease (NAFLD) | 7 | 151 | 0.7010877 | 9.58E-01 |
| ko04970 | Salivary secretion | 4 | 89 | 0.7055903 | 9.58E-01 |
| ko05120 | Epithelial cell signaling in Helicobacter pylori infection | 3 | 68 | 0.7101339 | 9.58E-01 |
| ko04144 | Endocytosis | 13 | 275 | 0.7130735 | 9.58E-01 |
| ko05164 | Influenza A | 8 | 174 | 0.7163069 | 9.58E-01 |
| ko00900 | Terpenoid backbone biosynthesis | 1 | 23 | 0.7165399 | 9.58E-01 |
| ko01040 | Biosynthesis of unsaturated fatty acids | 1 | 23 | 0.7165399 | 9.58E-01 |
| ko03060 | Protein export | 1 | 23 | 0.7165399 | 9.58E-01 |
| ko04977 | Vitamin digestion and absorption | 1 | 23 | 0.7165399 | 9.58E-01 |
| ko04650 | Natural killer cell mediated cytotoxicity | 6 | 133 | 0.7193211 | 9.58E-01 |
| ko04664 | Fc epsilon RI signaling pathway | 3 | 69 | 0.7194564 | 9.58E-01 |
| ko00340 | Histidine metabolism | 1 | 24 | 0.7316851 | 9.58E-01 |
| ko00592 | alpha-Linolenic acid metabolism | 1 | 24 | 0.7316851 | 9.58E-01 |
| ko00280 | Valine, leucine and isoleucine degradation | 2 | 48 | 0.7334885 | 9.58E-01 |
| ko00520 | Amino sugar and nucleotide sugar metabolism | 2 | 48 | 0.7334885 | 9.58E-01 |
| ko04920 | Adipocytokine signaling pathway | 3 | 71 | 0.7374017 | 9.58E-01 |
| ko04330 | Notch signaling pathway | 2 | 49 | 0.743909 | 9.58E-01 |
| ko04930 | Type II diabetes mellitus | 2 | 49 | 0.743909 | 9.58E-01 |
| ko00062 | Fatty acid elongation | 1 | 25 | 0.7460231 | 9.58E-01 |
| ko00982 | Drug metabolism - cytochrome P450 | 3 | 72 | 0.7460287 | 9.58E-01 |
| ko00564 | Glycerophospholipid metabolism | 4 | 95 | 0.7527844 | 9.58E-01 |
| ko05110 | Vibrio cholerae infection | 2 | 50 | 0.7539793 | 9.58E-01 |
| ko05320 | Autoimmune thyroid disease | 2 | 50 | 0.7539793 | 9.58E-01 |
| ko04918 | Thyroid hormone synthesis | 3 | 73 | 0.7544286 | 9.58E-01 |
| ko04145 | Phagosome | 7 | 160 | 0.7563852 | 9.58E-01 |
| ko01200 | Carbon metabolism | 5 | 119 | 0.7674958 | 9.65E-01 |
| ko04966 | Collecting duct acid secretion | 1 | 27 | 0.7724471 | 9.65E-01 |
| ko00591 | Linoleic acid metabolism | 1 | 28 | 0.7846122 | 9.65E-01 |
| ko00601 | Glycosphingolipid biosynthesis - lacto and neolacto series | 1 | 28 | 0.7846122 | 9.65E-01 |
| ko04520 | Adherens junction | 3 | 77 | 0.7858139 | 9.65E-01 |
| ko04310 | Wnt signaling pathway | 6 | 144 | 0.7868412 | 9.65E-01 |
| ko04914 | Progesterone-mediated oocyte maturation | 4 | 100 | 0.7874996 | 9.65E-01 |
| ko05130 | Pathogenic Escherichia coli infection | 2 | 55 | 0.7993485 | 9.69E-01 |
| ko04915 | Estrogen signaling pathway | 4 | 103 | 0.8063968 | 9.69E-01 |
| ko05166 | HTLV-I infection | 11 | 256 | 0.8109089 | 9.69E-01 |
| ko04742 | Taste transduction | 3 | 81 | 0.813812 | 9.69E-01 |
| ko00640 | Propanoate metabolism | 1 | 32 | 0.8271217 | 9.69E-01 |
| ko03410 | Base excision repair | 1 | 32 | 0.8271217 | 9.69E-01 |
| ko05132 | Salmonella infection | 3 | 84 | 0.8327259 | 9.69E-01 |
| ko04110 | Cell cycle | 5 | 131 | 0.8348324 | 9.69E-01 |
| ko05034 | Alcoholism | 7 | 177 | 0.8400218 | 9.69E-01 |
| ko05202 | Transcriptional misregulation in cancers | 7 | 177 | 0.8400218 | 9.69E-01 |
| ko00561 | Glycerolipid metabolism | 2 | 61 | 0.8438995 | 9.69E-01 |
| ko00020 | Citrate cycle (TCA cycle) | 1 | 34 | 0.8451258 | 9.69E-01 |
| ko00051 | Fructose and mannose metabolism | 1 | 34 | 0.8451258 | 9.69E-01 |
| ko05020 | Prion diseases | 1 | 35 | 0.8534138 | 9.69E-01 |
| ko05330 | Allograft rejection | 1 | 35 | 0.8534138 | 9.69E-01 |
| ko05340 | Primary immunodeficiency | 1 | 35 | 0.8534138 | 9.69E-01 |
| ko03018 | RNA degradation | 3 | 88 | 0.8553628 | 9.69E-01 |
| ko00350 | Tyrosine metabolism | 1 | 36 | 0.8612595 | 9.69E-01 |
| ko03030 | DNA replication | 1 | 36 | 0.8612595 | 9.69E-01 |
| ko00190 | Oxidative phosphorylation | 5 | 142 | 0.8817366 | 9.85E-01 |
| ko00830 | Retinol metabolism | 2 | 68 | 0.8844241 | 9.85E-01 |
| ko04211 | Longevity regulating pathway - mammal | 3 | 95 | 0.8885903 | 9.85E-01 |
| ko00620 | Pyruvate metabolism | 1 | 41 | 0.8946337 | 9.85E-01 |
| ko04976 | Bile secretion | 2 | 71 | 0.8986194 | 9.85E-01 |
| ko00040 | Pentose and glucuronate interconversions | 1 | 42 | 0.900278 | 9.85E-01 |
| ko04152 | AMPK signaling pathway | 4 | 125 | 0.9067205 | 9.85E-01 |
| ko00970 | Aminoacyl-tRNA biosynthesis | 1 | 44 | 0.910678 | 9.85E-01 |
| ko04962 | Vasopressin-regulated water reabsorption | 1 | 44 | 0.910678 | 9.85E-01 |
| ko04142 | Lysosome | 4 | 130 | 0.9217897 | 9.85E-01 |
| ko01230 | Biosynthesis of amino acids | 2 | 77 | 0.9222718 | 9.85E-01 |
| ko03420 | Nucleotide excision repair | 1 | 47 | 0.9242849 | 9.85E-01 |
| ko04380 | Osteoclast differentiation | 4 | 131 | 0.9245285 | 9.85E-01 |
| ko03015 | mRNA surveillance pathway | 3 | 108 | 0.932716 | 9.89E-01 |
| ko03022 | Basal transcription factors | 1 | 51 | 0.939266 | 9.89E-01 |
| ko03460 | Fanconi anemia pathway | 1 | 51 | 0.939266 | 9.89E-01 |
| ko05012 | Parkinson's disease | 4 | 141 | 0.9475286 | 9.93E-01 |
| ko00230 | Purine metabolism | 5 | 177 | 0.9633511 | 1.00E+00 |
| ko00500 | Starch and sucrose metabolism | 1 | 62 | 0.9669004 | 1.00E+00 |
| ko05131 | Shigellosis | 1 | 65 | 0.9719551 | 1.00E+00 |
| ko05322 | Systemic lupus erythematosus | 3 | 130 | 0.9726705 | 1.00E+00 |
| ko05016 | Huntington's disease | 5 | 188 | 0.9753567 | 1.00E+00 |
| ko00010 | Glycolysis / Gluconeogenesis | 1 | 71 | 0.9798711 | 1.00E+00 |
| ko04660 | T cell receptor signaling pathway | 2 | 112 | 0.9846604 | 1.00E+00 |
| ko03008 | Ribosome biogenesis in eukaryotes | 1 | 83 | 0.9896399 | 1.00E+00 |
| ko03013 | RNA transport | 3 | 169 | 0.9950384 | 1.00E+00 |
| ko03040 | Spliceosome | 1 | 138 | 0.999514 | 1.00E+00 |
| ko03010 | Ribosome | 1 | 147 | 0.9997062 | 1.00E+00 |
| ko04740 | Olfactory transduction | 8 | 391 | 0.999744 | 1.00E+00 |
