# Supplementary material for: Novel mutations of TCTN3/LTBP2 with cellular function changes in congenital heart disease associated with polydactyly
Source: J Cell Mol Med. 2020 Oct 24;24(23):13751–62. doi: 10.1111/jcmm.15950 (PMC7753982; doi:10.1111/jcmm.15950)
Supplement: Supplementary file 8 — Supplementary Material [file JCMM-24-13751-s008.doc]

**Supplementary method**

**Whole Exome Sequencing (WES) and validated by Sanger sequencing**

***WES research***

One blood sample was obtained from the case and extracted gDNA. The study protocol was approved by the Institutional Review Board and Ethics Committee of TICH.

WES was performed at the BGI Company (BGI Clinical Laboratories, Shenzhen, china). Through sequencing of the samples and analyzing the SNV/Indel detected, we aim to find the genetic reasons that maybe relevant with disease in the patient. The WES results were validated by sanger sequencing.

***Verified mutation sites by sanger sequencing***

Genomic DNA was extracted from the peripheral leukocytes of the patient and control subject. Respectively, two pairs of primers were designed to amplify fragments on both sides of the two gene mutation sites (TC*TN3* c.1268G>A, *LTBP2* c.2206G>A) in patient and control. The PCR products were sequenced by 3500 Genetic Analyzer (Applied Biosystems) directly to verify the results of WES. The PCR primers and conditions are described in supplementary table 1. The same sets of primers were used for PCR and bi-directional sequencing.

**Establishment of human pluripotent stem cell lines with mutations
*Vector construction***

To verify the correlation between TCTN3/LTBP2 mutation and clinical phenotype of the patient in vivo, human pluripotent stem cells (hPSCs) with point mutation was used as model by CRISPR/Cas9-mediated genome engineering ([Ran et al,2013](https://www.nature.com/articles/nprot.2013.143)).

The plasmid pSpCas9(BB)-2A-Puro(PX459) was a gift from Feng Zhang (Addgene plasmid no. 48139). Single-guide RNAs (sgRNAs) were designed using the online design tool available at http://crispr.mit.edu. Pairs of oligos including targeting sequences were annealed and cloned into the BbsI site of the Cas9 expression plasmid px459. The double-stranded DNA was used as repair template. Templates were generated by pEASY-Blunt Simple Cloning Kit (CB111-01, TransGen Biotech) according to manufacturer’s protocol. Null sense mutations on templates were generated to avoid double strands break by Cas9. Primers used can be found in supplementary table1.

Human pluripotent stem cell culture and transfection

The hPSC lines were cultured in Essential 8 medium (E8, A1517001, Thermo Fisher Scientific) without feeder. Culture dishes and plates were coated with Matrigel (356230, Corning) for one hour at 37℃ incubator before seeding cells. The medium was changed daily and cells routinely passaged every 4 days.

Cells were cultured to reach confluency of 80%, transfection was performed using the Human Stem Cell Nucleofector Kit 1 (VPH-5012 Lonza) and the Amaxa Nucleofector Ⅱ according to manufacturer’s protocol. Cells were washed with PBS twice, dissociated by EDTA and resuspended in E8 medium supplemented with 10 μM ROCK inhibitor (Rocki). Transfection solution was prepared by adding 10μL supplement to 90μL Human Stem Cell Nucleofector solution 1 and 10μg plasmid per round of nucleofection. One sixth of resuspended cells was centrifuged and the pellet was suspended by mixed transfection solution and transferred to the cuvette without air bubbles and the Amaxa Nucleofector Ⅱand program B-016 was executed. Nucleofected cells were retrieved by pipettes and added to E8 with 10 μM Rocki in Matrigel coated plate.

***Puromycin selection***

About 24 hours after transfection, E8 medium was replaced selected by 2μg/ml puromycin. Approximately six hours later, medium was changed to regular E8 medium to stop selection and subsequently changed daily after about 5 days, hPSCs colonies were separated and cut into two pieces by 10 μL pipette, one piece was transferred into the PCR tube containing DNA lysis buffer and the other was move into a new well on a Matrigel coated 24-well plate. DNA products were amplified by PrimeSTAR Max Premix (R045, TaKaRa). PCR amplified was carried out using PrimeSTAR Max Premix (R045, TaKaRa) and the cycle condition was 95℃ for 5min, followed by 35 cycles at 95℃ for 30s, 58℃ (LTBP2) or 62℃ (TCTN3) for 30s and 72℃ for 50s. Sanger sequencing were used to test mutations. Positive clones were further cultured for following experiments. Primers used can be found in supplementary table 1. The hPSC lines were used as control group.

**Detection of the developmental pluripotency of hPSCs.**

***Western Blot***

Cells were lysed in cell lysis buffer on ice for 30 minutes and sonicated for 1 minute at 60 amplitude at 2 seconds intervals. Samples were centrifuged at 10,000g for 10 minutes at 4℃. Then supernatants were transferred into new pre-cooled tubes. The protein concentration was measured by BCA Protein Assay Kit (23227, Thermo Fisher Scientific), and then the samples were boiled together with SDS buffer at 98℃ for 8 minutes. 20μg protein of each sample was separated on 8% or 10% Bis-Tris SDS-PAGE and transferred to polyvinylidene difluoride (PVDF) membranes. Membranes were incubated in 5% skim milk in TBST at room temperature for 2 hours and probed with primary antibodies at 4℃ overnight, including anti-OCT4 (sc5279, Santa Cruz), anti-Nanog (sc293121, Santa Cruz), anti-TNNT2 (ab45932, Abcam), anti-ACTN2 (A7811, Sigma), anti-β-actin (P30002, Abmart). Immunoreactive bands were then incubated for 2 hours at room temperature with appropriate horseradish peroxidase (HRP)-conjugated secondary antibodies, including anti-Rabbit IgG-HRP (NA934V, GE Healthcare) and goat anti-Mouse IgG (H+L)/HRP (ZB-2305, ZSGB-BIO). Target protein bands were detected by Chemiluminescent HRP substrate (WBKLS0500, Millipore).

***Quantitative real-time PCR***

Total RNA was extracted using TRIzol (15596018, Thermo Fisher Scientific) according to manufacturer’s protocol. The first-strand cDNA was synthesised from 2 μg RNA using random primers (Roche) and M-MLV Reverse Transcriptase. Real-time quantitative PCR reactions were set up in duplicate with the FS Universal SYBR Green Master (Roche) and carried out in an iCycler MyiQ2 Detection System (BIO-RAD). Each sample was repeated 3 times and normalized using GAPDH as internal control. The amplification was performed for primary denaturation at 95℃ for 10 minutes, then 40 cycles of denaturation at 95℃ for 15s, annealing and elongation at 58℃ for 1min, and the last cycle under 55-95℃ for dissociation curve. Relative quantitative evaluation of target gene was determined by comparing the threshold cycles. Primers were confirmed for the specificity with dissociation curves. Primers used can be found in supplementary table 1.

***Immunofluorescence Microscopy***

The cells were washed twice in PBS and fixed in freshly prepared 3.7% paraformaldehyde for 30 minutes on ice, then washed once in PBS and permeabilized in 0.1% Triton X-100 for 30 minutes at room temperature, followed by blocking using 3% goad serum and 0.1% BSA for 2 hours at room temperature. Samples were incubated with the primary antibodies overnight at 4℃. The primary antibodies used include anti-OCT4, anti-Nanog, anti-SSEA4 (MAB4303, Millipore), anti-TNNT2, anti-ACTN2. After washed three times in PBS, the samples were incubated with secondary antibody at room temperature for two hours using Goat Anti-Mouse IgG (H+L) FITC (115–095-003; Jackson), Goat Anti-Rabbit IgG (H+L) Alexa Fluor® 594 (111–585-003; Jackson) and Goat Anti-Mouse IgM Alexa Fluor® 488(A-21042; Invitrogen). The cell nucleus were stained for 10 minutes with DAPI, and placed in Vectashield mounting medium. Fluorescence was detected and imaged by Carl Zeiss Axio-Imager Z2 fluorescence microscope.

**Differentiation of hPSCs into cardiomyocytes**

The PSC Cardiomyocyte Differentiation Kit (A2921201, Thermo Fisher Scientific) was used for differentiation of hPSCs into cardiomyocytes according to manufacturer’s protocol. Briefly, the cells were cultured in A medium for first two days and B medium for two days, then changed to maintenance medium. The medium was changed every other day. Cell samples were collected on day9 and day13 during differentiation.

**RNA-sequencing and bioinformatics analysis**

Cardiomyocyte cells derived from hPSCs were cultured and harvested. A total amount of 3 mg of RNA per sample was extracted. The mRNA was enriched and fragmented into short fragments and reverse transcripted into cDNA with random primers. Sequencing libraries were generated using NEBNext Ultra RNA Library Prep Kit for Illumina (NEB, United States) following manufacturer’s recommendations and index codes were added to attribute sequences to each sample. The sequencing was performed at the Gene Denovo Biotechnology Company (Guangzhou, China).

After total RNA was extracted, Then the enriched mRNA was fragmented into short fragments using fragmentation buffer and reverse transcripted into cDNA with random primers. Second-strand cDNA were synthesized by DNA polymerase I, RNase H, dNTP and buffer. Then the cDNA fragments were purified with QiaQuick PCR extraction kit, end repaired, poly(A) added, and ligated to Illumina sequencing adapters. The ligation products were size selected by agarose gel electrophoresis, PCR amplified, and sequenced using Illumina HiSeqTM 2500 by Gene Denovo Biotechnology Co. (Guangzhou, China).

For bioinformatics analysis, clean reads were mapped to Genome Reference Consortium Human Build 38 (GRCh38.p10). Gene abundances were quantified by software RSEM. R software was used to perform principal component analysis (PCA) and identify differentially expressed genes (DEGs) between samples and control (<http://www.r-project.org/>).

DEGs expression pattern analysis was performed to assess the gene expression trend during the development of myocardial cell, using Short Timeseries Expression Miner (STEM) software ([Ernst J, 2006](https://www.ncbi.nlm.nih.gov/pubmed/16597342)). Then DEGs were subjected to enrichment analysis of Gene Ontology (GO) functions and KEGG (Kyoto Encyclopedia of Genes and Genomes) pathways ([Kanehisa M, 2017](https://www.ncbi.nlm.nih.gov/pubmed/?term=KEGG%3A+new+perspectives+on+genomes%2C+pathways%2C+diseases+and+drugs)).

**Statistical analysis**

Data were analyzed by ANOVA or t-tests using StatView software from SAS Institute Inc. The P value was calculated, statistically significance was defined as P < 0.05(*), P < 0.01(**) or P < 0.001(***).
